# Supplementary figures and images for: Different disease inoculations cause common responses of the host immune system and prokaryotic component of the microbiome in Acropora palmata
Source: PLoS One. 2023 May 25;18(5):e0286293. doi: 10.1371/journal.pone.0286293 (PMC10212133; doi:10.1371/journal.pone.0286293)

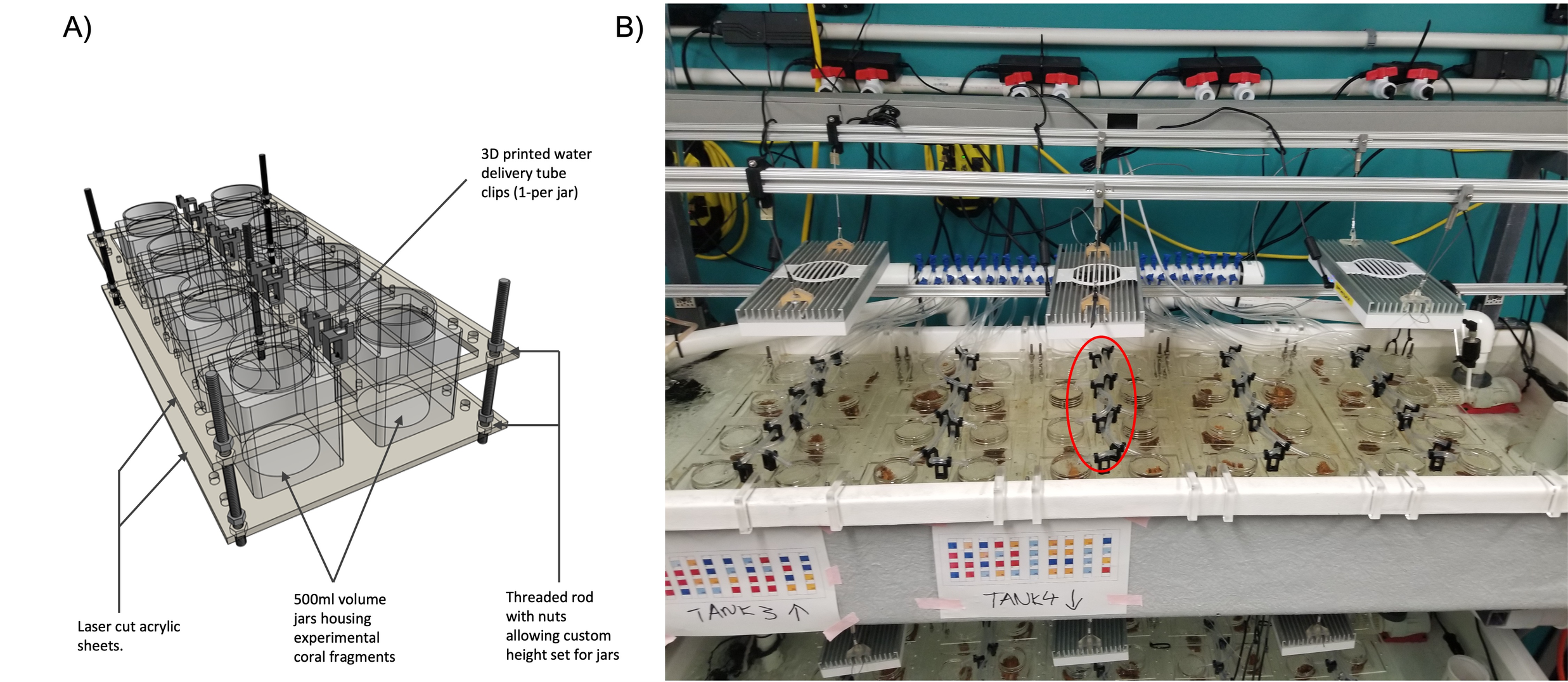

Supplement: S1 Fig — A) Computer aided design model of the disease rack setup. Each rack housed eight experimental jars with each jar receiving a dedicated water supply secured via 3D printed clips. Each raceway held five of these racks resulting in 40 experimental jars per raceway. Jar lip was above the raceway water level allowing high replication within one raceway minimizing potential tank effect as well as mitigating cross-contamination between experimental fragments. B) Example of a raceway with five of the disease racks shown in part A). Dedicated 3D printed water clips providing water flow to each jar are shown in red circle for one rack. Experimental A. palmata fragments are visible within the experimental jars. (TIF) [file pone.0286293.s001.tif]

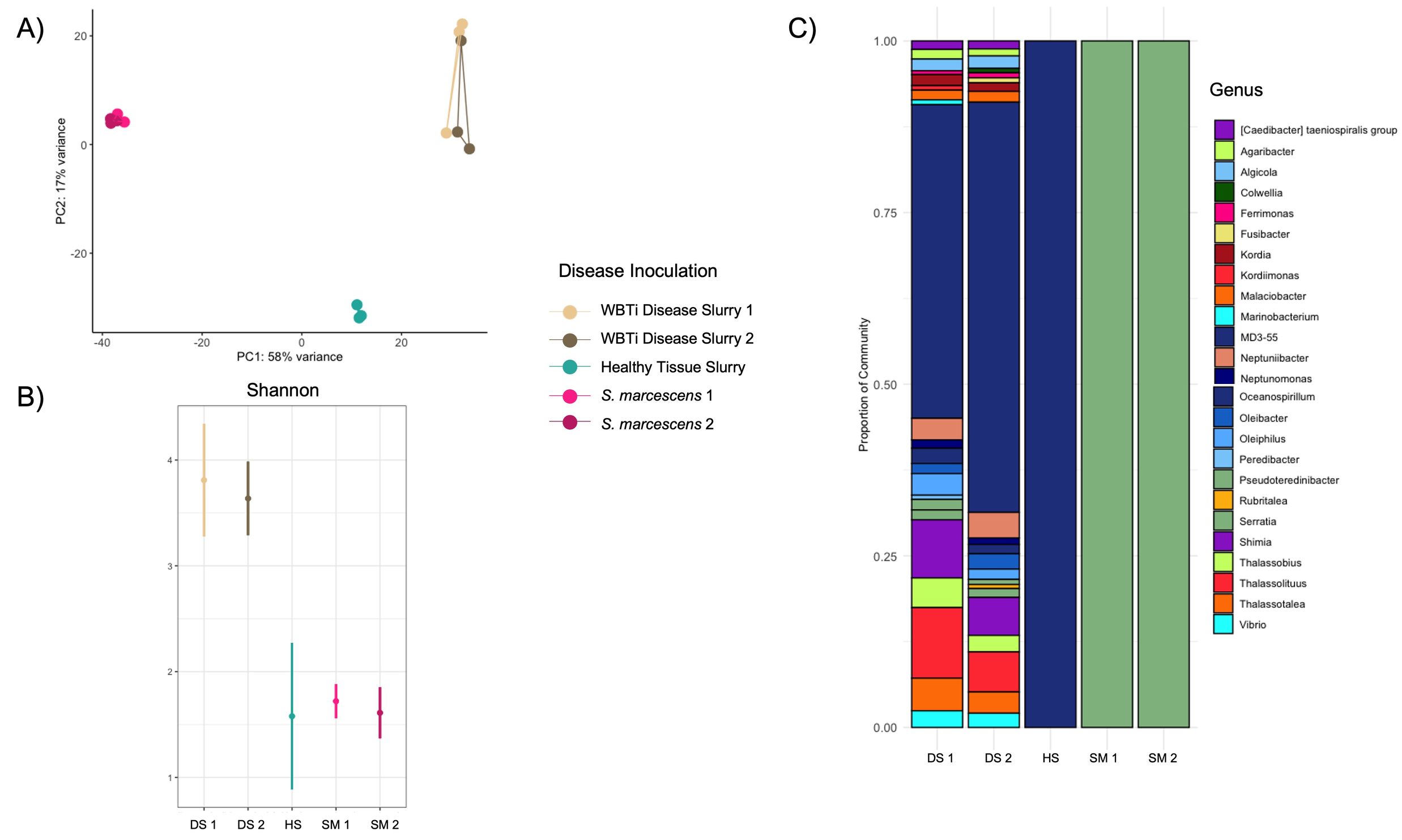

Supplement: S2 Fig — A) Principal component [PC] analysis showed separation of the different disease inoculations. PC1 (59%) identified differences between the slurry inoculations (WBTi Disease Slurry [WBTi DS], Healthy Tissue Slurry [HTS]) and the S. marcescens [SM] inoculations. PC2 (17%) identified a difference between the pathogenic inoculations (WBTi DS and SM) and the HTS. B) Shannon-Weiner alpha diversity estimates (y-axis) identified more diverse microbiomes in the WBTi DS inoculations compared to the HTS and SM inoculations. X-axis shows the different disease inoculations. C) Relative abundance analysis of the inoculation samples, at the genus level, with genus with <0.05 abundance removed. Y-axis shows proportions of each genus contributing to relative abundance. Legend to right indicates the fill to genus color for the stacked bar plot. For B) and C): DS1 = WBTi Disease slurry inoculation 1. DS2 = WBTi Disease Slurry inoculation 2. HTS = Healthy Tissue Slurry. SM1 = S. marcescens inoculation one. SM2 = S. marcescens inoculation two. (TIF) [file pone.0286293.s002.tif]

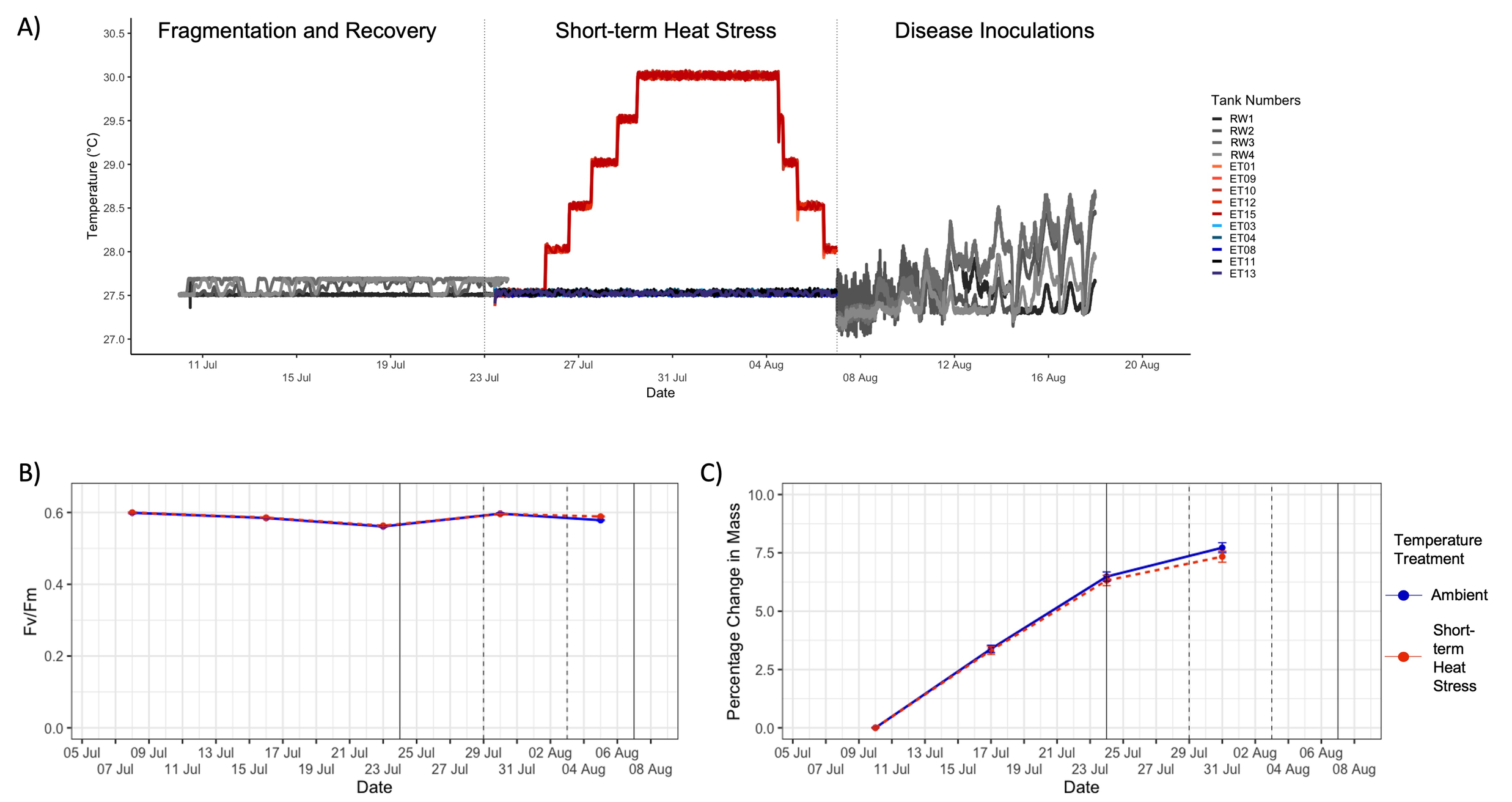

Supplement: S3 Fig — A) Temperature data was collected from temperature loggers in the four raceways and ten experimental tanks. Lines plotted are for all the raceways and experimental tanks. The first section (label = “Fragmentation and Recovery) shows the period after fragmentation allowing healing of all wounds. Temperature was set to 27.5°C. The middle section (labeled = Short-term Heat Stress [STHS]) shows the 15-day temperature stress run in 10 experimental tanks. Ambient corals remained at 27°C (blue lines). STHS corals experienced a 5-day ramp of 0.5°C/day, 5-days at 30°C, and a 5-day ramp back down to 27°C (0.5°C/day). The final section (labeled Disease Inoculations) shows temperature of the raceway with disease rack set up. Temperature was set to 27°C but due to the jar set up within the four raceways, temperature showed larger fluctuation than during the Fragmentation and Recovery period. Temperature fluctuations were roughly +/-1°C. RW = Raceways (with tanks denoted by numbers 1 to 4), and ET = experimental tanks. STHS tanks are denoted by numbers 1, 9, 10, 12, 15, and red lines. Ambient tanks are denoted by numbers 3, 4, 8, 11, 13, and blue lines. B) Calculated Fv/Fm values for all genets split in ambient (blue line) and STHS (red line). There were no significant differences between ambient or STHS at any of the measurement timepoints (alpha < 0.05). C) Calculated increase in mass for all genet fragments in the ambient (blue line) and STHS (red line) treatments. Percentage change was utilized due to the surface area not being taken of all fragments in the experiment. There were no significant differences between either treatment at any of the measured timepoints. For B) and C), X-axes = Date. Solid vertical lines in plots indicate the start (24th July) and end (7th August) of the STHS portion of the experiment. Dotted lines indicate the start (29th July) and end (3rd August) of the 5-days spent at 30°C for corals experiencing the STHS. (TIF) [file pone.0286293.s003.tif]

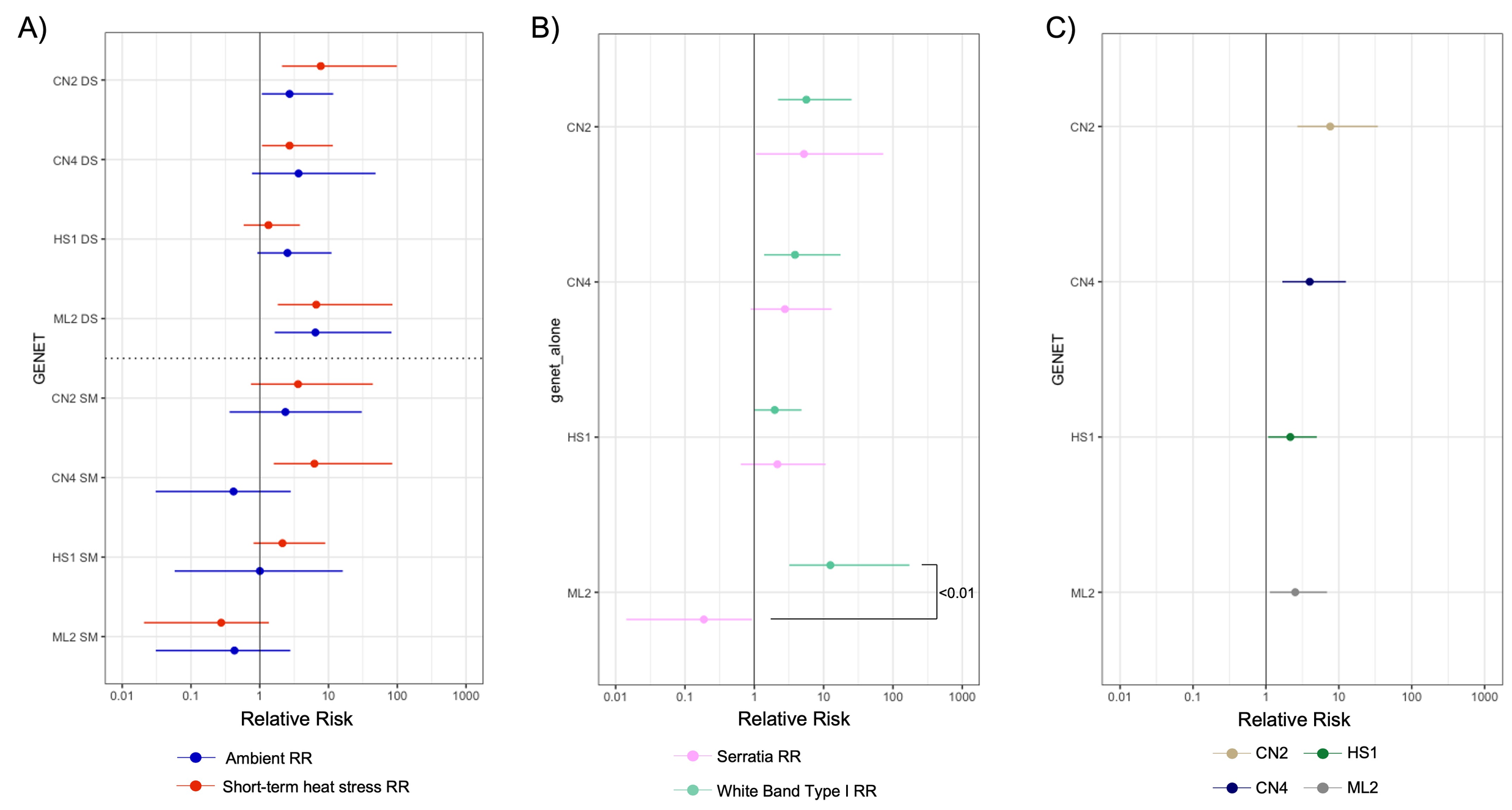

Supplement: S4 Fig — A) Relative risk [RR] analysis of the effect of temperature treatment on risk for each genet within each pathogenic treatment. There were no significant effects of tank temperature on the relative risk for each genet within the Disease Slurry RR (WBTi Disease Slurry [WBTi DS] versus healthy tissue slurry [HTS]) or the Serratia RR analysis (Serratia marcescens [SM] versus Serratia placebo [SP]). Y axis = genets (CN2, CN4, HS1, ML2) followed by inoculation type; DS = relative risk of WBTi DS vs HTS, SM = relative risk of SM vs SP. Blue = median risk of each fragment in ambient temperature treatments. Red = median risk of fragments in the short-term heat stress [STHS] temperature treatment. B) Analysis of the Disease Slurry RR (WBTI DS versus healthy HTS) or Serratia RR (SM versus SP) for each genet. There were no differences in risk between the Disease Slurry RR and Serratia RR for genets CN2, CN4, and HS1. There was a significant difference between the relative risks for genet ML2 (alpha < 0.01). Y axes = genets (CN2, CN4, HS1, ML2) with each genet split by: Pink = median risk of each genet for the Serratia RR, light green = median risk of each genet for the calculated Disease Slurry RR. C) RR analysis grouping all disease inoculations into either pathogenic (WBTi DS and SM) or control (HTS) and SP). There were no significant differences for Pathogenic RR between any of the genets. Y-axis = Genets (CN2, CN4, HS1, ML2) with genet color identified below plot. For A), B) and C), the Bayesian relative risk analysis, on a log scale, was used for different subsets of the data. Lines depict the 95% credible intervals of the Bayesian analysis. X-axes = log scale. Y-axes = each respective plots variable. 95% confidence intervals that are positive and do not cross 1 indicate a significant higher risk for the pathogenic inoculation than the control inoculation. 95% confidence intervals that are less than 1 indicate a significant lower risk for pathogenic inoculations than the c [file pone.0286293.s004.tif]

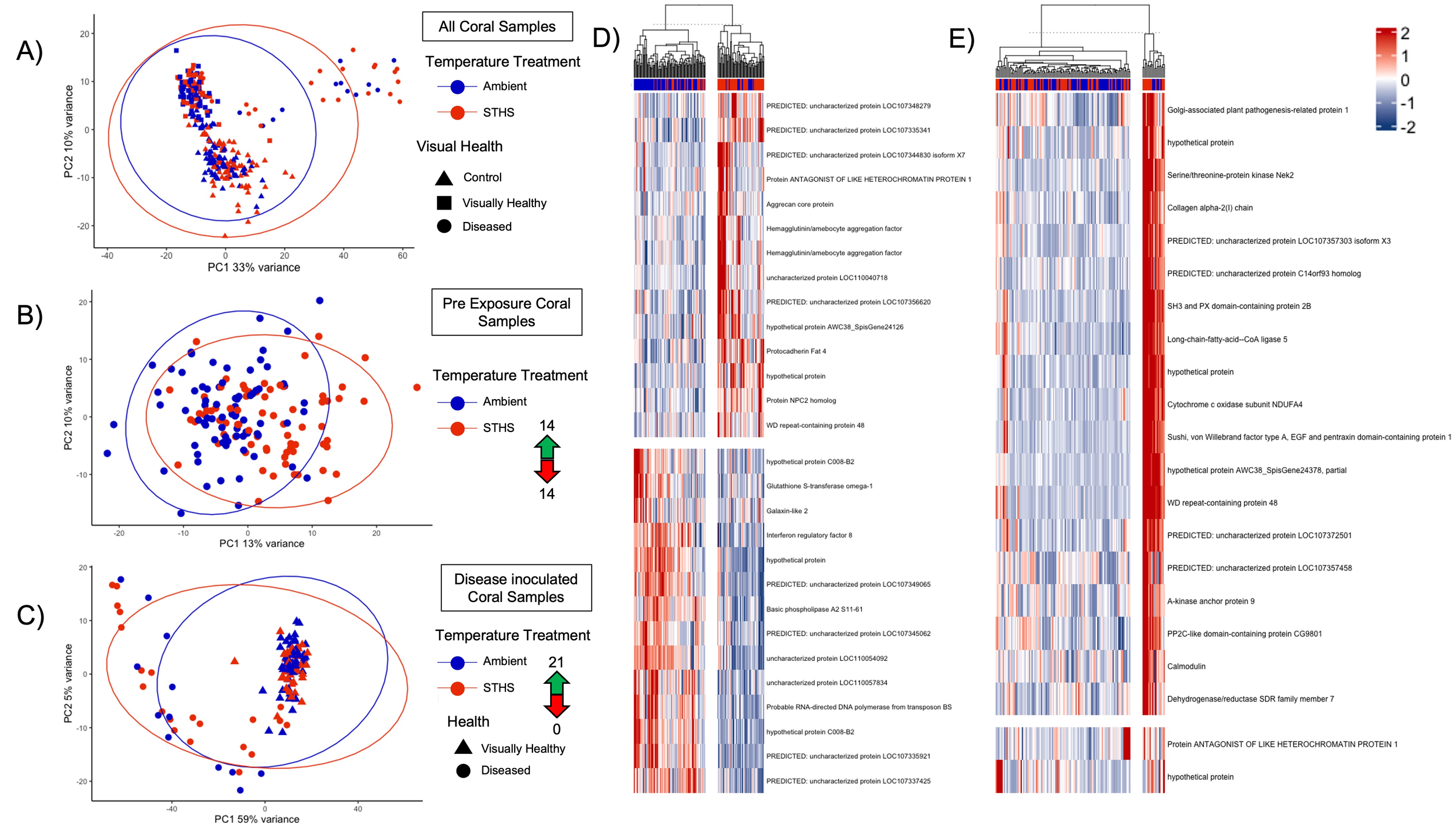

Supplement: S5 Fig — A) All coral samples (control, visually healthy and diseased) showed no clear separation of 95% confidence intervals between ambient and short-term heat stressed [STHS] corals. B) Pre-exposure coral samples showed no clear separation of 95% confidence intervals between the STHS and the ambient temperature treatments in PC analysis. Differential expression analysis identified 12 significantly upregulated and 12 significantly downregulated genes. C) Disease inoculated coral samples showed no clear separation of 95% confidence intervals between the STHS and ambient temperature treatments in PC analysis. Differential expression only identified 21 significantly upregulated genes. D) Heatmap showing the 28 significantly differentially expressed genes identified for the pre-exposure coral samples. Heatmap was generated using the variance stabilized transformed [VST] counts of pre-exposure coral samples. Hierarchical clustering of columns (pre-exposure coral samples, dendrogram shown) and rows (genes, dendrogram not shown) identified clustering by temperature treatment with some overlap. E) Heatmap showing the 21 significantly differentially expressed genes identified from the disease inoculated coral samples. Heatmap was generated using the VST counts of disease inoculated coral samples. Hierarchical clustering of columns (disease inoculated coral samples, dendrogram shown) and rows (genes, dendrogram not shown) identifies large overlap between STHS and ambient corals. For A), B), and C), the VST counts with genet variance removed was used. Blue dots = ambient corals (maintained at 27˚C). Red dots = STHS corals (5-days at 30˚C). X-axes show principal component 1. Y-axes show principal component 2. For B) and C), green arrow shows significantly upregulated genes (alpha < 0.01, L2FC <-1) and red arrow shows significantly downregulated genes (alpha < 0.01, L2FC <-1). For A) and C), shapes show visual health status [VHS] of each coral sample. (TIF) [file pone.0286293.s005.tif]

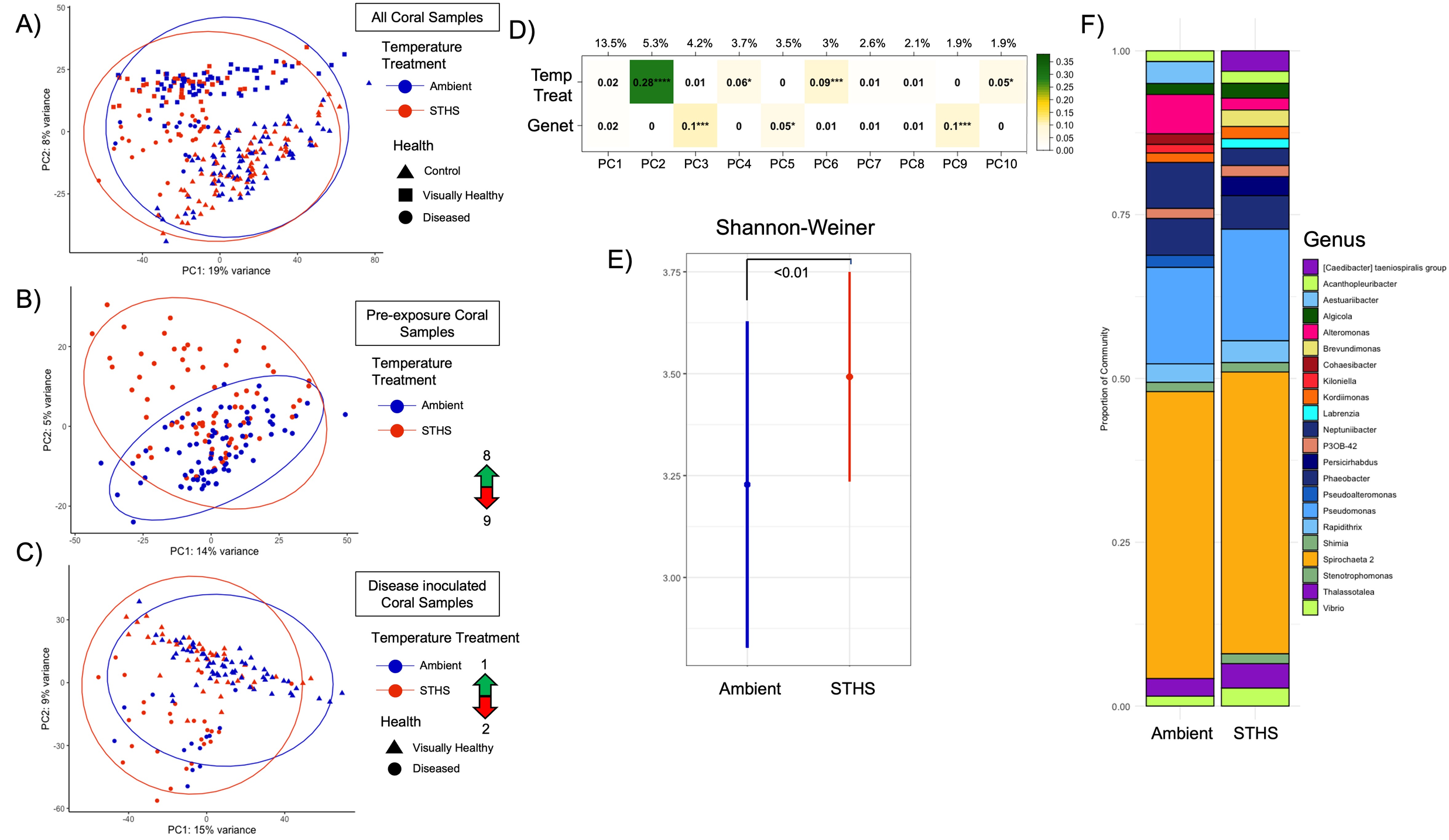

Supplement: S6 Fig — A) Principal component [PC] analysis of all coral samples identified no clear separation of 95% confidence intervals due to temperature treatment. B) PC analysis of pre-exposure coral samples identified stronger grouping of ambient compared to short-term heat stress coral samples, with this being significant in PERMANOVA analysis. C) PC analysis identified no clear separation of disease inoculated coral samples due to temperature treatment. D) Correlative analysis between PCs and traits (temperature treatment and genet identity) interest for pre-exposure coral samples. R2 correlation is shown within each heatmap cell with fill showing stronger correlations (green) to weaker correlations (yellow to white). Number of stars within heatmap cells shows the significance of R2 correlations to principal component and metadata variable (* = <0.05, ** = <0.01, *** = <0.001, **** = < 0.0001). E) Shannon-Weiner alpha diversity estimates between ambient and short-term heat stress [STHS] for pre-exposure corals. Points are the Shannon-Weiner alpha metric mean estimates with bars showing calculated confidence intervals. There was a significant difference (alpha < 0.01) between ambient and STHS stress pre-exposure coral samples in the Shannon-Weiner alpha diversity estimates. F) Relative abundance analysis, to the genus level, between ambient and STHS pre-exposure coral samples. Genera with < 0.01 average abundance were excluded from visualization. Genera bar color fills are shown in legend to right of plot. For A), B), and C), the center logged transformed [CLR] ASV counts were used for each subset of data. Blue dots = ambient corals (maintained at 27˚C). Red dots = STHS corals (5-days at 30˚C). X-axes show principal component 1. Y-axes show principal component 2. For B) and C), green arrow shows significantly increased abundance ASVs (alpha < 0.01, LFC <-1) and red arrow shows significantly decreased abundance ASVs (alpha < 0.01, LFC <-1). For A) and C), shapes show visual health [file pone.0286293.s006.tif]

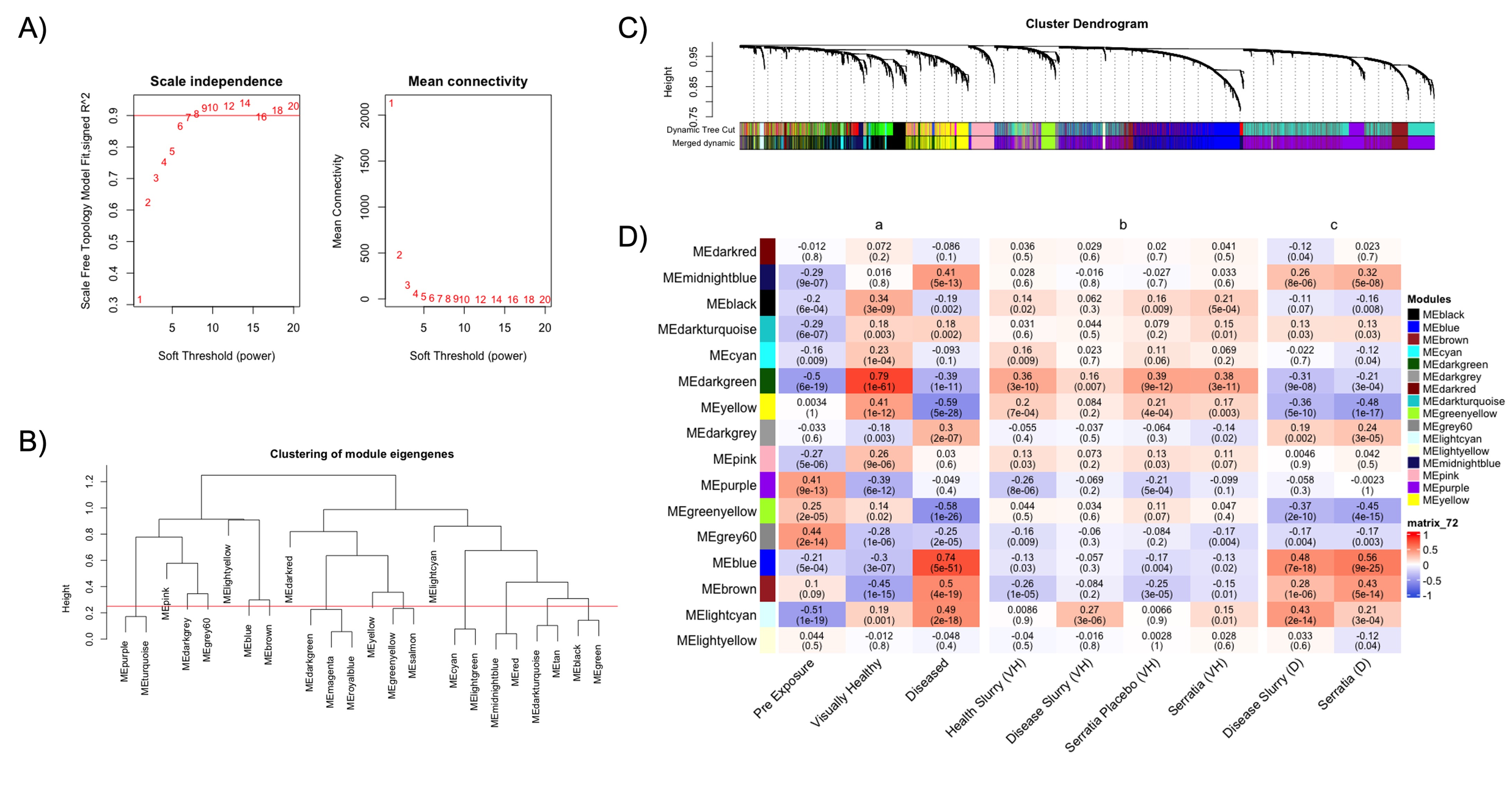

Supplement: S7 Fig — A) Scale independence and mean connectivity plots from WGCNA pipeline identifying a soft power of 7 which was used in adjacency matrix calculations. B) Hierarchical clustering of identified module eigengenes with the red horizontal line indicating the cut height (0.25) for merging of modules. C) Cluster dendrogram showing the dynamic tree height with pre-merged modules (Dynamic Tree Cut) and post-merged modules (Merged Dynamic). D) Full module to metadata correlation heatmap for all 16 modules identified through WGCNA analysis. Modules are rows and generic color name is specified to the left of heatmap. Columns are metadata traits split into: a) grouped visual health status (pre-exposure, visually healthy, and diseased), b) visually healthy corals split by disease inoculations (healthy tissue slurry [HTS], WBTi disease slurry [WBTi DS], Serratia placebo [SP], and Serratia marcescens [SM]), and c) diseased coral samples spilt by disease inoculation (WBTi DS, and SM). Heatmap fill shows positive correlations (red) and negative correlations (blue). For each cell, the upper value identifies module correlation with metadata trait, lower value shows significance of the correlation. (TIF) [file pone.0286293.s007.tif]

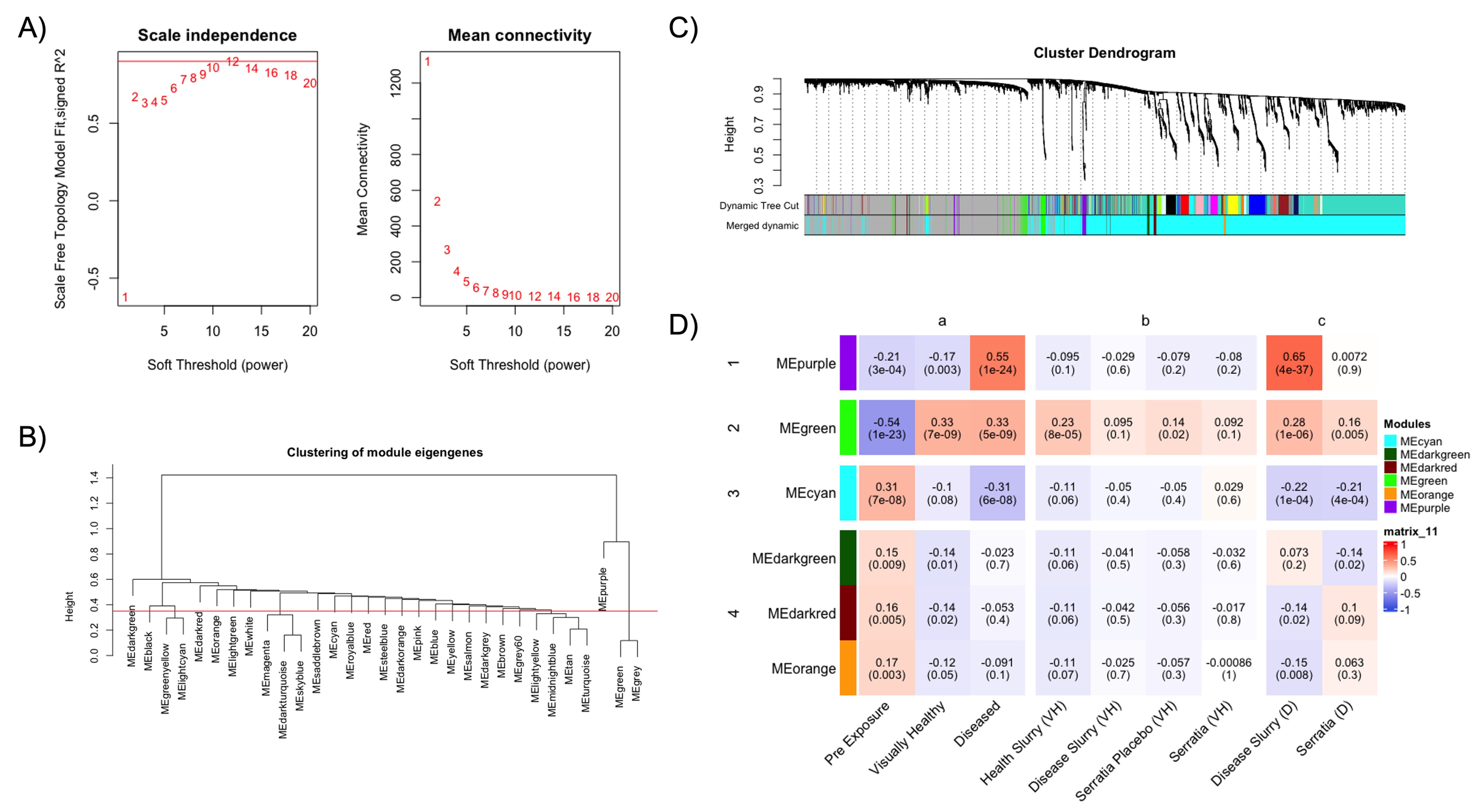

Supplement: S8 Fig — A) Scale independence and mean connectivity plots from WGCNA pipeline identifying a soft power of 12 which was used in adjacency matrix calculations. B) Hierarchical clustering of identified module eigengenes with the red horizontal line indicating the cut height (0.35) for merging of modules. C) Cluster dendrogram showing the dynamic tree height with pre-merged modules (Dynamic Tree Cut) and post-merged modules (Merged Dynamic). D) Full module to metadata correlation heatmap for all 6 modules identified through WGCNA analysis. Modules are rows and generic color name is specified to the left of heatmap. The “Grey” module was removed from this plot. Columns are metadata traits split into a) grouped visual health status [VHS] (pre-exposure, visually healthy, and diseased), b) visually healthy corals split by disease inoculations (healthy tissue slurry [HTS], WBTi disease slurry [WBTi DS], Serratia placebo [SP], and Serratia marcescens [SM]), and c) diseased coral samples spilt by disease inoculation (WBTi DS, and SM). Heatmap fill show positive correlations (red) and negative correlations (blue). For each cell, upper value identifies module correlation with metadata trait, lower value shows significance of the correlation. (TIF) [file pone.0286293.s008.tif]

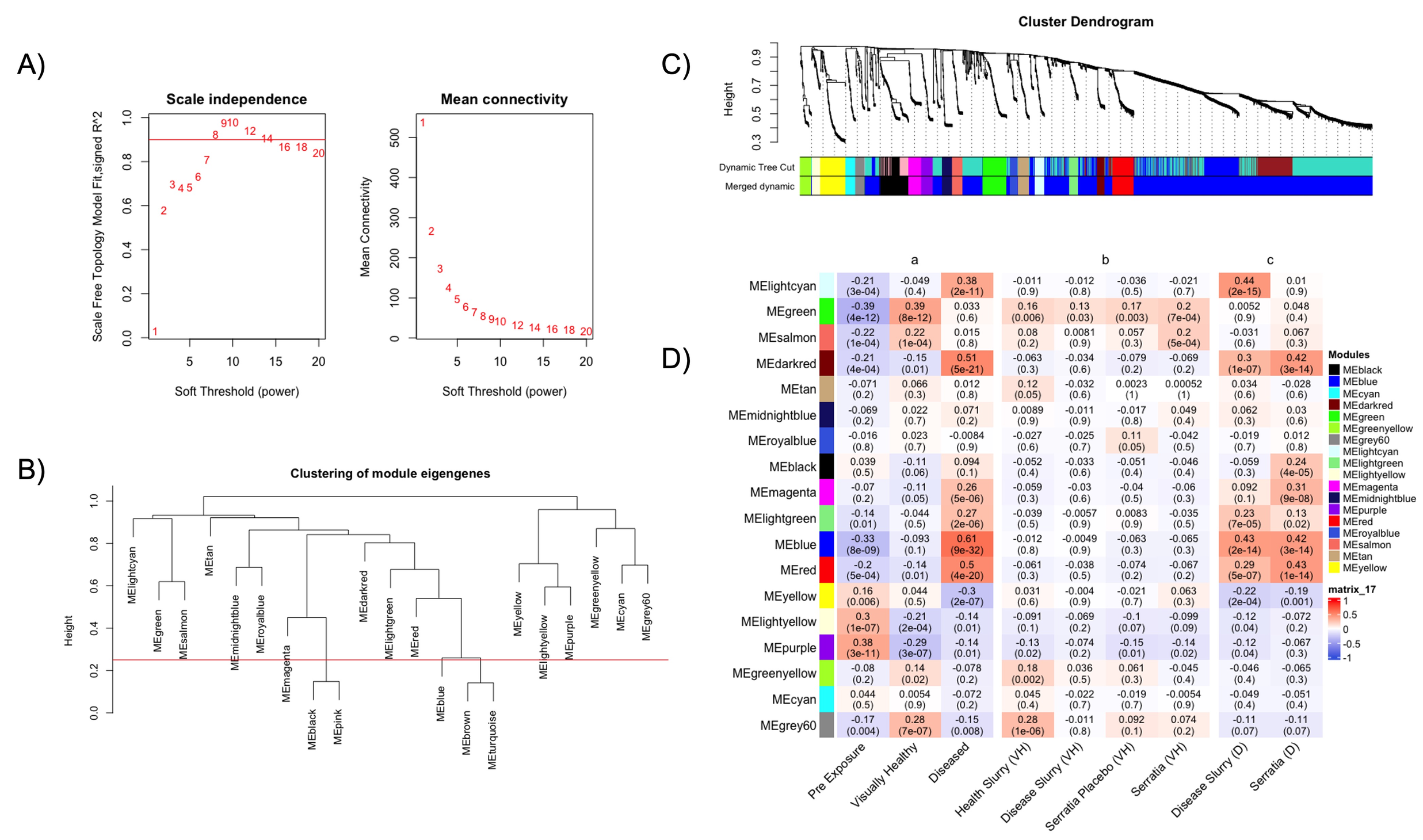

Supplement: S9 Fig — A) Scale independence and mean connectivity plots from WGCNA pipeline identifying a soft power of 8 which was used in adjacency matrix calculations. B) Hierarchical clustering of identified module eigengenes with the red horizontal line indicating the cut height (0.25) for merging of modules. C) Cluster dendrogram showing the dynamic tree height with pre-merged modules (Dynamic Tree Cut) and post-merged modules (Merged Dynamic). D) Full module to metadata correlation heatmap for the 18 modules identified through WGCNA analysis. Modules are rows and generic color name is specified to the left of heatmap. Columns are metadata traits split into a) grouped visual health status [VHS] (pre-exposure, visually healthy, and diseased), b) visually healthy corals split by disease inoculations (healthy tissue slurry [HTS], WBTi disease slurry [WBTi DS], Serratia placebo [SP], and Serratia marcescens [SM]), and c) diseased coral samples spilt by disease inoculation (WBTi DS, and SM). Heatmap fill show positive correlations (red) and negative correlations (blue). For each cell, top value identifies module correlation with metadata trait, bottom value shows significance of the correlation. (TIF) [file pone.0286293.s009.tif]

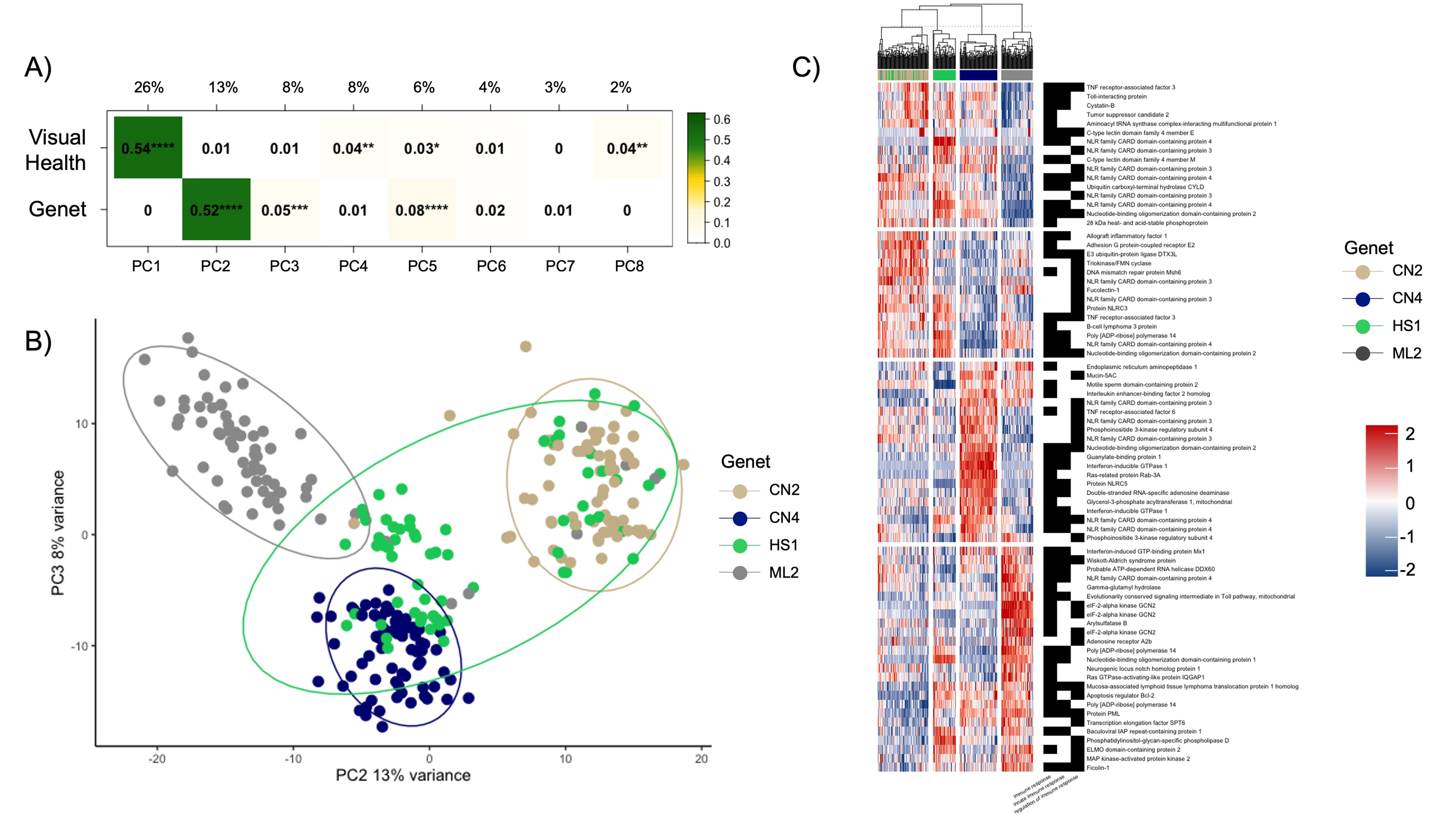

Supplement: S10 Fig — A) Correlation matrix of the genet and visual health status [VHS] variables and their correlations to identified principal components [PC]. R2 correlation is shown within each heatmap cell with fill showing stronger correlations (green) to weaker correlations (yellow to white). Number of stars within heatmap cells shows the significance of R2 correlations to PC and metadata variable (Genet, VHS). Stars signify significance levels, * = <0.05, ** = <0.01, *** = <0.001, **** = < 0.0001. B) Visualization of PC2 and PC3 showed strong clustering by genet identity. Circles are all coral samples (control, visually healthy, and diseased). Ellipsis show the 95% confidence intervals calculated for samples within each genet. C) Left Heatmap shows the 75 most significant genes, identified from the likelihood ratio test [LRT] in DeSeq2, linked to immune GO terms from GO enrichment analysis. Main heatmap was generated using the variance stabilized transformed [VST] counts. Hierarchical clustering of columns (coral samples, dendrogram shown) and rows (genes, dendrogram not shown) was performed. Hierarchical clustering of the columns (coral samples) showed strong clustering of genet identity for genet ML2 (dark grey), CN4 (dark blue), and CN2 (wheat). Genet HS1 (green) also generated a cluster but with inclusion of samples from the other genets. Hierarchical clustering of genes (rows) also identified expression clusters for specific genets. Right heatmap is a presence (black fill) absence (white fill) of genes to the three identified immune GO terms from GO enrichment analysis: Immune Response (GO:0006955), Regulation of Immune Response (GO:0050776), and Innate Immune Response (GO:0045087). (TIF) [file pone.0286293.s010.tif]

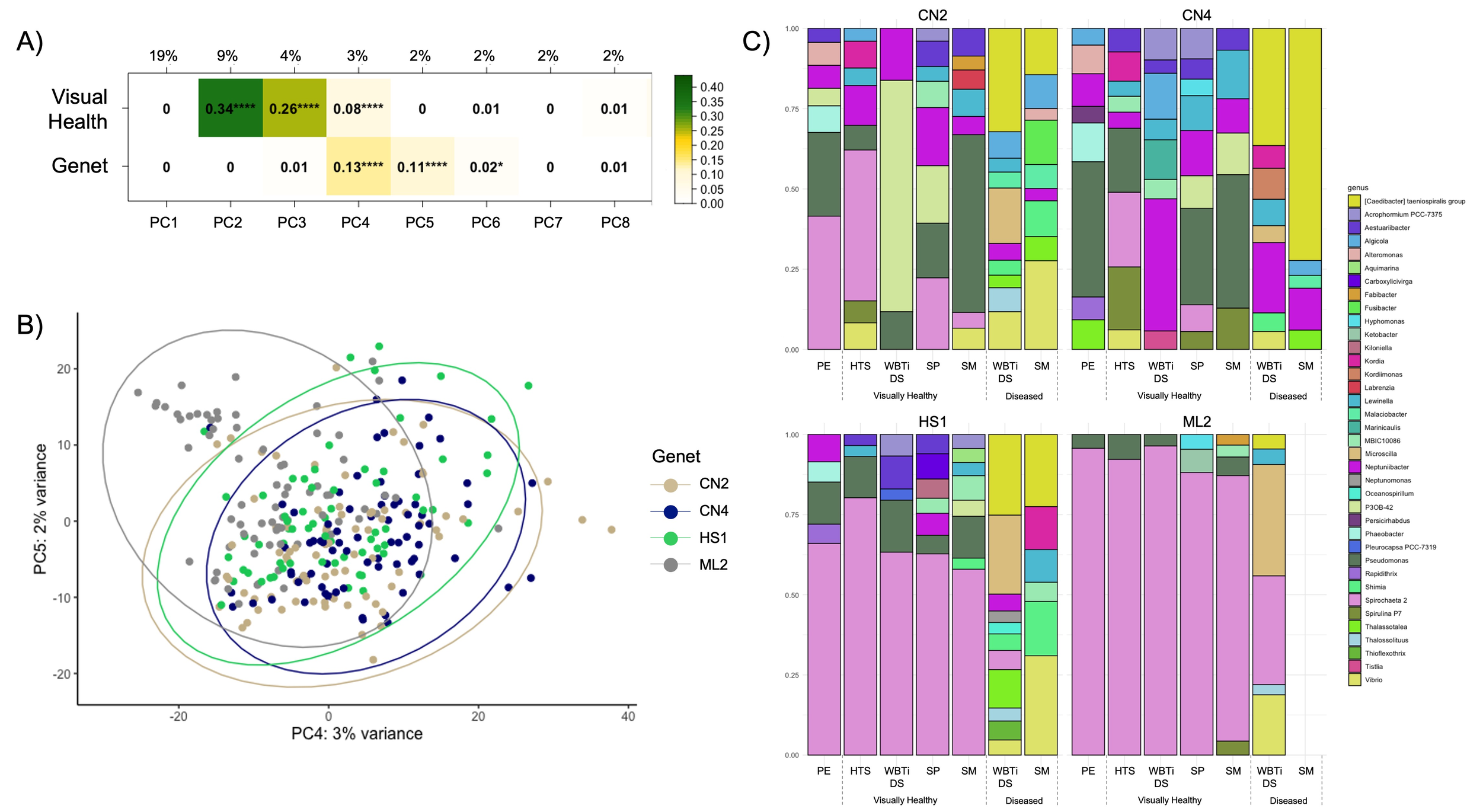

Supplement: S11 Fig — A) Correlation matrix of principal components [PC] 1 to PC8 to the visual health status [VHS] and genet identity metadata variables. Correlation of metadata trait to PC is shown in each cell, with fill showing stronger correlations (green) to weaker correlations (yellow to white). Number of stars within heatmap cells shows the significance of R2 correlations to principal component and metadata variable (* = <0.05, ** = <0.01, *** = <0.001, **** = < 0.0001). B) Visualization of PC4 (3% variance) and PC5 (2% variance) due to significant correlations of genet through correlative analysis. Samples are colored only by genet with legend to the right of plot. C) Relative abundance analysis for each genet split by VHS and disease inoculations. Genera with <0.025 average abundance were excluded from visualization. Top left plot = CN2, top right plot = CN4, bottom left plot = HS1, bottom right plot = ML2. For each genet plot, x-axis shows: PE (pre-exposure), visually healthy coral samples split by disease inoculation (HTS = healthy tissue slurry, WBTi DS = white band type I disease slurry, SP = Serratia placebo, SM = Serratia marcescens), and diseased corals split by disease inoculations (WBTi DS = white band type I disease slurry, SM = Serratia marcescens). For each pot y-axes show relative proportion. For all plots, bar fills follow the legend to the right showing genus to color fill. (TIF) [file pone.0286293.s011.tif]
