# Supplementary material for: Different disease inoculations cause common responses of the host immune system and prokaryotic component of the microbiome in Acropora palmata
Source: PLoS One. 2023 May 25;18(5):e0286293. doi: 10.1371/journal.pone.0286293 (PMC10212133; doi:10.1371/journal.pone.0286293)
Supplement: S1 File — (DOCX) [file pone.0286293.s012.docx]

# Chlorophyll Measurements

Imaging Pulse Amplitude Modulation fluorometry (I-PAM) was used to assess the health of the coral fragments via photochemical efficiency of the dinoflagellate endosymbiont (1) throughout the initial recovery and temperature exposure phases of the experiment. This resulted in five measurement timepoints for photochemical efficiency. Corals were dark-adapted for one hour in ERL before measurements were taken. I-PAM measurements were taken on the Symbiodiniaceae denser sun-facing side of the fragments which was identified at the fragmentation stage. I-PAM measurements were taken at the tip, center, and bottom of each *A. palmata* fragment allowing a whole fragment average to be taken. The Fv/Fm was calculated using the Fm and F values as follows:

$$FvFm=\frac{Fm-F}{Fm}$$

I-PAM results were analyzed to look at the effect of the short-term heat stress using analysis of variance (ANOVA) and a posthoc Tukey’s HSD test. The model *FvFM ~ Temperature Exposure * IPAM Sampling Point* was used to identify any significant changes between I-PAM measurements due to temperature treatments at each sampling point. Alpha was set at 0.05. Visualization of Fv/Fm values was done in GGplot (Wickham, 2016) with the averaged Fv/Fm values for fragments in each temperature exposure: ambient (27ºC), and short-term heat stress (STHS) (5-days at 30ºC), averaged for plotting.

# Buoyant Weight

Buoyant weight of fragments was measured to assess the health of the fragments during the recovery and temperature exposure phases of the experiment. This resulted in four measurement timepoints for buoyant weight. The buoyant weight setup in the ERL was used and followed the methods of [2] using a PA22c Pioneer (Ohaus) scale. All fragments were weighed in their respective tank water at the same water temperature in an acrylic cube with a shield to reduce fragment sway due to air movement. Water temperature was maintained and kept within 0.2ºC of 27.5ºC (recovery period and ambient fragments) and 30ºC (short-term heat stress fragments). The acrylic plugs on which fragments were attached were cleaned prior to weighing to remove any weight addition from biofouling agents. Prior and post-fragment weighing, water samples were taken from the acrylic cube to calculate dry weight. Water samples were run through the DMA 5000 M densitometer (Anton Paar), which provided the salinity. Salinity and temperature were then used to calculate density and fragment dry weight.

For each timepoint taken, percentage growth was calculated. An arcsine transformation was used on the percentage change data to meet ANOVA assumptions. The model *arcsine percentage change ~ Temperature Exposure * Buoyant Weight Sampling Point* was run, and a posthoc Tukey HSD test was used to identify any significant differences between ambient or short-term heat-stressed corals at each buoyant weight sampling point. Alpha was set to 0.05. Visualization of the buoyant weight was done in GGplot [3] with percentage change data averaged for fragments in each temperature exposure: ambient (27ºC), and short-term heat stress (STHS) (5-days at 30ºC), averaged for plotting.

# Relative Risk Analysis

Relative risk (RR) analyses were run to compare the chances of disease signs occurring for different subsets of the *A. palmata* fragments. This included 1) identifying how temperature treatment for each genet affected disease incidence within the WBTi DS and HTS, and within the *SM* and *S*P, 2) risk of disease occurring between the four genets without temperature treatment included for WBTi DS and HTS, and *SM* and *S*P, and 3) for the four genets without including temperature treatment and pooling pathogenic inoculations (WBTi DS and *SM*) and non-pathogenic inoculations (HTS and *S*P). The remainder of the RR methods uses the analysis of 1) above: identifying how temperature treatment for each genet affected disease incidence within the WBTi DS and HTS, and within the *SM* and *S*P.

For the RR a Bayesian analysis was used as it allows inference from the posterior distribution to identify significant increases or decreases in the risk ratio. To calculate RR, the risk of disease was calculated as the proportion of diseased to total population (diseased/total population) for risk inoculated (WBTi DS or *SM*) and non-risk inoculated (HTS or *S*P) fragments within each genet (CN1, CN2, ML2, HS1) and temperature treatment (ambient or STHS). The RR was then calculated for each genet in each temperature exposure (ambient or short-term heat stress) and resulted in a WBTi RR analysis (WBTi DS = risk inoculation, HTS = no risk inoculation), and a *SM* RR analysis (*SM* = risk inoculation, S*P =* no risk inoculation):

$$Relative Risk=\frac{Risk in Exposed}{Risk in non-exposed}$$

When RR=1, there is no association between risk exposure and occurrence of disease. A RR>1 indicates a positive association with the risk exposure, whereas a RR<1 indicates a negative association with the risk exposure. The posterior distribution of each RR was calculated using a Bayesian approach [4] with a binomial likelihood distribution and a uniform-Beta prior distribution. Estimates of RR and 95% credible intervals were obtained by using a Markov Chain Monte Carlo simulation with Gibbs sampling in the R package R2jag [5]. Visualization of the RR and 95% confidence intervals was done in GGplot [3]. To test whether there were significant differences due to temperature exposure (ambient or STHS) between each genet within the WBTi RR analysis and *SM* RR analysis, the methodology presented in (6) was followed. Briefly, the log values of each RR and 95% confidence intervals were calculated, and the standard error of each log RR was obtained by dividing the width of the confidence interval by 2 x 1.96. The difference between pairs of log RR and standard errors was then calculated, before being divided to test interaction. Significance for the interaction was tested (stats::pnorm) with key parameters as follows: mean = 0, sd = 1, lower.tail = F. This allowed pairwise analysis and significance testing of the effect of temperature exposure (ambient versus STHS) on the calculated RR and 95% confidence intervals for each genet in each disease RR analysis (WBTi RR analysis, and *SM* RR analysis).

# Nucleic Acid Extractions

Each coral tissue sample was placed in a Zymo bead beating tube (0.1mm and 0.5mm beads) filled with 750µl Zymo DNA/RNA shield. Bead beating tubes where then placed on a horizontal vortex at max power for 40 minutes. Following manufacturer recommendations, a max of 24 bead beating tubes were placed in the vortex attachment so to maintain a high speed and maximum bead beating efficiency. Samples then followed the adapted automation Zymo RNA/DNA MagBead extraction protocol for the Kingfisher Flex (Thermofisher). Briefly, each bead beating tube was centrifuged at 14,000g for 30 seconds and 200µl supernatant was added to a Kingfisher Flex deep well plate. 10µl of reconstituted proteinase K was then added to each well, mixed, and incubated at room temperature for 30 minutes. After incubation, 500µl of DNA/RNA lysis buffer, and 30µl Zymo MagBinding beads were added to each well. The deep well plate was then loaded onto the Kingfisher instrument and followed the Kingfisher Zymo DNA parallel protocol. After the first binding step, the original sample plate was removed, sealed and placed on ice ready for RNA processing on the Kingfisher Flex. On completion of the Kingfisher Zymo DNA parallel protocol, the eluted DNA in a deep well plate was removed from the instrument, sealed and placed at -80^º^C. 350µl from the original deep well sample plate was transferred to a new Kingfisher deep well plate, and 350µl of 95-100% molecular grade ethanol, and 30µl of Zymo MagBinding Beads were added to each well. The Kingfisher Zymo RNA Parallel protocol was then initiated. During the Kingfisher Zymo RNA parallel protocol, the recommended 20-minute DNase was included as recommended in manufacturer protocol. On completion of the Kingfisher Zymo RNA parallel protocol, the eluted RNA was removed from the Kingfisher Flex, and 10µl aliquoted to a new plate for quality control processing. Both plates were then sealed and placed at -80ºC. Please also see [7] for Kingfisher scripts and step-by-step protocol.

# 16S rRNA Library Preparation

For amplification, each sample was processed in a 50µl reaction with the following reagents: 2x Platinum Hot Start PCR Master Mix (20µl), PCR-grade water (26µl), 10µm reverse (1µl), and forward primer (1µl) and sample DNA (2µl). For negative controls 2µl of PCR-grade water was used. DNA was then amplified using the following thermocycler parameters: 94^o^C for 3 minutes (1x), 94 ^o^C for 45 seconds (35x), 50 ^o^C for 60 seconds (35x), 72 ^o^C for 90 seconds (35x), and 72 ^o^C for 10 minutes (1x). All PCR products were visualized on a 1.2% agarose gel. PCR products were cleaned using 60µl of AMPure XP beads (Beckham Coulter, Brea, CA) and 80% ethanol before being resuspended in PCR-grade water. Cleaned samples were then quantified using the High Sensitivity DNA Assay Kit (Thermofisher) and a Qubit v3, normalized to 4nM in PCR-grade water, and 5µl of each sample pooled for sequencing.

# 3` RNA-seq Pre-processing

Demultiplexing was undertaken by the sequencing facility. Raw reads were QC’ed using FastQC [8], and adapter sequences trimmed using BBduk.sh from BBtools [9] using the recommended parameters from Lexogen (<https://www.lexogen.com/quantseq-data-analysis/>). Trimmed reads were then aligned to the *A. palmata* transcriptome [10,11] using Salmon [12] and alignment rates were checked with STAR [13]. Aligned samples were imported into RStudio, using tximport [14], and a transcript-to-gene file generated from the *A. palmata* genome GFF3 file [10,11], allowing quantification to the gene level. Samples with low sequencing depth (<1,000,000 counts across all genes), and genes with less than two counts in greater than 20 samples, were removed.

# 16S rRNA pre-processing

MiSeq sequences were demultiplexed by the sequencing facility, and PCR primers were removed from the forward and reverse reads using Cutadapt v3.4 [15]. To infer ASVs, the DADA2 [16] pipeline was run on each MiSeq run independently. Reads were trimmed and filtered (dada2::filterAndTrim; truncLen = c(210,140), minLen = 100, trimLeft =10) based on quality profiles plots (dada2::plotQualityProfile). Error models were generated for each MiSeq runs forward (FWD) and reverse (REV) reads separately (dada2::learnErrors) before being visualized (dada2::plotErrors). Each lane's FWD and REV reads were dereplicated (dada2::derepFastq) and amplicon sequence variants (ASVs) inferred using the dereplicated and generated error models (dada2::dada). FWD and REV inferred ASVs were merged (dada2::mergePairs) and a count table generated for each identified ASV (dada2::makeSequenceTable). Chimeras were removed (dada2::removeBimeraDenovo) and taxonomy was assigned (dada2::IdTaxa) to ASVs using the SILVA_SSU_r138_2019 reference database [17]. The data from each run was then combined. Prior to analysis, the data underwent QC using phyloseq v1.34.0 [18]. Samples with ASV counts of <2000, and ASVs with taxonomic identification to mitochondrial, chloroplast, or Eukaryotes (using SILVA, generated phylogenetic tree, and BLASTn) were removed. Finally, ASVs with <10 counts across all coral samples were filtered and used for downstream analyses.

# 16S rRNA Classification of N/A ASVs

Due to the number of uncultured marine bacterium [19,20] and unknown nature of the majority of coral disease pathogens [21-23], all ASVs that were not assigned taxonomy using the SILVA database were retained for further classification. ASVs not assigned to the genus level were entered into a local search (NCBI Blast+ v2.12.0) using the nucleotide (nt) database (v6) with following parameters; task = megablast, max_target_seqs = 5, max_hsps = 5, word_size 5. Taxonomizer v0.8.0 [24] was used to provide full taxonomies for the blast results using the taxonomic IDs, and a generated SQLite database of all taxonomic assignments on the NCBI (taxonomizr::prepareDatabase). Full taxonomy for results was then obtained (taxonomizr::getTaxonomy). ASVs which had successful BLAST hits with the assigned parameters were then used to update the taxonomy file generated in the DADA2 pipeline.

A phylogenetic tree was constructed for all ASVs identified from DADA2 using the r package phangorn v2.7.1 [25]. A nearest joining tree was generated (phangorn::treeNJ) before model parameters were optimized (phangorn::optim.pml), then visualized in FigTree v1.4.4 (<http://tree.bio.ed.ac.uk/software/figtree/>). Tree inspection then allowed putative annotation of unknown ASVs to differing taxonomic levels depending on the surrounding successfully annotated ASVs using SILVA and the local Blast search. ASVs which were annotated by either of these methods are denoted “PUTATIVE”.

# Bibliography S1 File

1. Ralph PJ, Hill R, Doblin MA, Davy SK. Theory and Application of Pulse Amplitude Modulated Chlorophyll Fluorometry in Coral Health Assessment. In: Woodley CM, Downs CA, Bruckner AW, Porter JW, Galloway SB, editors. Diseases of Coral [Internet]. Hoboken, NJ: John Wiley & Sons, Inc; 2015 [cited 2022 Dec 20]. p. 506–23. Available from: https://onlinelibrary.wiley.com/doi/10.1002/9781118828502.ch38

2. Dodge RE, Wyers SC, Frith HR, Knap AH, Smith SR, Cook CB, et al. Coral calcification rates by the buoyant weight technique: Effects of alizarin staining. J Exp Mar Biol Ecol. 1984 Mar;75(3):217–32.

3. Wickham H. ggplot2: Elegant Graphics for Data Analysis [Internet]. 2016. Available from: https://ggplot2.tidyverse.org.

4. Lawson AB. Bayesian Disease Mapping: Hierarchical Modeling in Spatial Epidemiology. [Internet]. 3rd ed. Vol. 1. Chapman and Hall; 2018. 486 p. Available from: https://doi.org/10.1201/9781351271769

5. Su YS, Yajima M. Packlage R2jags: A packacge for running jags from R. 2012.

6. Altman DG. Statistics Notes: Interaction revisited: the difference between two estimates. BMJ. 2003 Jan 25;326(7382):219–219.

7. Young BD. Automated DNA/RNA Extractions from a stony coral (Acropora palmata) using ZymoBIOMICS DNA/RNA Magbead Kit and the Kingfisher Flex v2 [Internet]. 2022 Jul [cited 2022 Nov 4]. Available from: https://www.protocols.io/view/automated-dna-rna-extractions-from-a-stony-coral-a-ccqnsvve

8. Andrews S. FastQC: A Quality Control Tool for High Throughput Sequence Data [Internet]. 2010. Available from: http://www.bioinformatics.babraham.ac.uk/projects/fastqc/

9. Bushnell B. BBTools [Internet]. 2014. Available from: https://jgi.doe.gov/data-and-tools/software-tools/bbtools/bb-tools-user-guide/

10. Kitchen SA, Aakrosh Ratan, Bedoya-Reina O, Burhans R, Fogarty ND, Webb Miller, et al. Genomic Variants Among Threatened Acropora Corals. 2019;9(1633):31919109 Bytes.

11. Baums IB. Baums Laboratory | Penn State University Marine Biology | Research in Coral Ecology and Evolution [Internet]. 2018 [cited 2022 Nov 9]. Available from: http://baumslab.org/

12. Patro R, Duggal G, Love MI, Irizarry RA, Kingsford C. Salmon provides fast and bias-aware quantification of transcript expression. Nat Methods. 2017 Apr;14(4):417–9.

13. Dobin A, Davis CA, Schlesinger F, Drenkow J, Zaleski C, Jha S, et al. STAR: ultrafast universal RNA-seq aligner. Bioinformatics. 2013 Jan;29(1):15–21.

14. Soneson C, Love MI, Robinson MD. Differential analyses for RNA-seq: transcript-level estimates improve gene-level inferences [version 2; peer review: 2 approved]. F1000 Res. 2016;4(1521):23.

15. Martin M. Cutadapt removes adapter sequences from high-throughput sequencing reads. EMBnet J. 2011 May;17(1):10–2.

16. Callahan BJ, McMurdie PJ, Rosen MJ, Han AW, Johnson AJA, Holmes SP. DADA2: High-resolution sample inference from Illumina amplicon data. Nat Methods. 2016 Jul;13(7):581–3.

17. Quast C, Pruesse E, Yilmaz P, Gerken J, Schweer T, Yarza P, et al. The SILVA ribosomal RNA gene database project: improved data processing and web-based tools. Nucleic Acids Res. 2012 Nov 27;41(D1):D590–6.

18. McMurdie PJ, Holmes S. phyloseq: An R Package for Reproducible Interactive Analysis and Graphics of Microbiome Census Data. Watson M, editor. PLoS ONE. 2013 Apr 22;8(4):e61217.

19. Steen AD, Crits-Christoph A, Carini P, DeAngelis KM, Fierer N, Lloyd KG, et al. High proportions of bacteria and archaea across most biomes remain uncultured. ISME J. 2019 Dec;13(12):3126–30.

20. Vartoukian SR, Palmer RM, Wade WG. Strategies for culture of ‘unculturable’ bacteria: Culturing the unculturable. FEMS Microbiol Lett. 2010 Apr 27;no-no.

21. Mera H, Bourne DG. Disentangling causation: complex roles of coral-associated microorganisms in disease: Disentangling coral disease causation. Environ Microbiol. 2018 Feb;20(2):431–49.

22. Muller EM, van Woesik R. Caribbean coral diseases: primary transmission or secondary infection? Glob Change Biol. 2012 Dec;18(12):3529–35.

23. Ainsworth TD, Kramasky-Winter E, Loya Y, Hoegh-Guldberg O, Fine M. Coral Disease Diagnostics: What’s between a Plague and a Band? Appl Environ Microbiol. 2007 Feb;73(3):981–92.

24. Sherill-Mix S. taxonomizr: Functions to Work with NCBI Accessions and Taxonomy [Internet]. 2021. Available from: https:://CRAN.R-project.org/package=taxonomizr

25. Schliep KP. phangorn: phylogenetic analysis in R. Bioinformatics. 2011 Feb 15;27(4):592–3.
