# Supplementary material for: Different disease inoculations cause common responses of the host immune system and prokaryotic component of the microbiome in Acropora palmata
Source: PLoS One. 2023 May 25;18(5):e0286293. doi: 10.1371/journal.pone.0286293 (PMC10212133; doi:10.1371/journal.pone.0286293)
Supplement: Attachment — Submitted filename: response_to_reviewers.docx [file pone.0286293.s025.docx]

**Editor Comments**

**Comment:** Thank you for submitting your manuscript to PLOS ONE. After careful consideration, we feel that it has merit but does not fully meet PLOS ONE’s publication criteria as it currently stands. Therefore, we invite you to submit a revised version of the manuscript that addresses the points raised during the review process.

I would recommend the authors to provide a careful and concise revision of the manuscript and also attract their attention to generate figures as per PloS one publication standard. The abstract needs a thorough readout and minor rewrite to make it clearer to the broader audience. The other issues raised by the reviewer 2 sounds reasonable to me.

**Response:** Thank you very much for all the comments and suggestions. We are very thankful for all time spent by the editor and the reviewers to improve the manuscript.

Re: the figures, the original submission utilized the PLOS PACE program which said all figures were up to journal standards. Saying this, we have resubmitted all figures through PACE, and they all meet requirements again with better resolution. Please let us know if there are any additional problems with the figures.

Re: abstract, we have re-read and edited the abstract to make it read better and be more applicable for a broader audience. This is included in the tracked changes manuscript.

We have also reviewed the entire manuscript and made it more concise in specific areas where possible. These changes can be seen in the tracked changes document. As noted in one of the responses to reviewer 2, this manuscript was originally double the length due to the large amount of data and analysis undertaken, and we spent considerable time cutting it to half the length which we feel still keeps adequate analysis depth while also being more concise and streamlined.

**Journal Requirements:**

When submitting your revision, we need you to address these additional requirements.

**Comment 1:** Please ensure that your manuscript meets PLOS ONE's style requirements, including those for file naming. The PLOS ONE style templates can be found at
[https://journals.plos.org/plosone/s/file?id=wjVg/PLOSOne_formatting_sample_main_body.pdf](https://nam10.safelinks.protection.outlook.com/?url=https%3A%2F%2Fjournals.plos.org%2Fplosone%2Fs%2Ffile%3Fid%3DwjVg%2FPLOSOne_formatting_sample_main_body.pdf&data=05%7C01%7Cbenjamin.young%40earth.miami.edu%7C42329e02adbe494f494208db24a292ea%7C2a144b72f23942d48c0e6f0f17c48e33%7C0%7C0%7C638144053217491384%7CUnknown%7CTWFpbGZsb3d8eyJWIjoiMC4wLjAwMDAiLCJQIjoiV2luMzIiLCJBTiI6Ik1haWwiLCJXVCI6Mn0%3D%7C3000%7C%7C%7C&sdata=eW2x9A%2F8PCYqQuUoHDjMFTBs%2F0qBHc5Kl073gyVkECE%3D&reserved=0) and
[https://journals.plos.org/plosone/s/file?id=ba62/PLOSOne_formatting_sample_title_authors_affiliations.pdf](https://nam10.safelinks.protection.outlook.com/?url=https%3A%2F%2Fjournals.plos.org%2Fplosone%2Fs%2Ffile%3Fid%3Dba62%2FPLOSOne_formatting_sample_title_authors_affiliations.pdf&data=05%7C01%7Cbenjamin.young%40earth.miami.edu%7C42329e02adbe494f494208db24a292ea%7C2a144b72f23942d48c0e6f0f17c48e33%7C0%7C0%7C638144053217491384%7CUnknown%7CTWFpbGZsb3d8eyJWIjoiMC4wLjAwMDAiLCJQIjoiV2luMzIiLCJBTiI6Ik1haWwiLCJXVCI6Mn0%3D%7C3000%7C%7C%7C&sdata=T7Wh0ZVgsw1k9fNCTZZhCfJAPTavflkZUtORHjNGoxQ%3D&reserved=0)

**Response:** Thank you for identifying that the original submission did not meet all style requirements. We have gone through the style templates, and all aspects for the manuscript submission are now in line with requirements.

**Comment 2:**. Thank you for stating the following financial disclosure:
 "ICE, Grant number 31252, NOAA Coral Reef Conservation Program. ICE provided experimental design expertise, lab resources, and manuscript comments and edits. NTK supported by NSF Grant 1951826 and Protect our Reefs Grant 2018-23. NTK helped plan the experiment, and provided direction on bioinformatic analysis, as well as manuscript comments and edits on drafts. The funders had no role in study design, data collection and analysis, decision to publish, or preparation of the manuscript. "

Please state what role the funders took in the study. If the funders had no role, please state: "The funders had no role in study design, data collection and analysis, decision to publish, or preparation of the manuscript.". If this statement is not correct you must amend it as needed.
Please include this amended Role of Funder statement in your cover letter; we will change the online submission form on your behalf.

**Response:** Thank you for identifying the problem with the financial disclosure. The funders took no role in the study, and we have incorporated your suggestion into the financial disclosure statement which will be added into the cover letter. Thank you for changing this for us.

**Comment 3:** We note that you have stated that you will provide repository information for your data at acceptance. Should your manuscript be accepted for publication, we will hold it until you provide the relevant accession numbers or DOIs necessary to access your data. If you wish to make changes to your Data Availability statement, please describe these changes in your cover letter and we will update your Data Availability statement to reflect the information you provide.

**Response:** Thank you for this comment. The NCBI project number was included in the original submission, as well as the github link to analysis scripts. The NCBI project will go live once the acceptance of the manuscript is received.

NCBI Project ID: PRJNA895002

Github: <https://github.com/benyoung93/resposne_of_acropora_palmata_to_disease_and_heat>

**Comment 4:** Please ensure that you include a title page within your main document. You should list all authors and all affiliations as per our author instructions and clearly indicate the corresponding author.

**Response:** Thank you for this comment. We have double checked the Plos ONE instructions for the title page and everything is now as required. We have removed zip codes, postal addresses, and street addresses in the affiliations section. States within the USA have also been written in full and not abbreviated.

We have also added in the corresponding author only including the email as stated on the style page. We have added the * to the respective author in the author list to make this clear.

**Comment 5:** Please review your reference list to ensure that it is complete and correct. If you have cited papers that have been retracted, please include the rationale for doing so in the manuscript text, or remove these references and replace them with relevant current references. Any changes to the reference list should be mentioned in the rebuttal letter that accompanies your revised manuscript. If you need to cite a retracted article, indicate the article’s retracted status in the References list and also include a citation and full reference for the retraction notice.

**Response:** Thank you for this comment. We have gone through our reference list and have found no indications of retracted articles. All references were screened on the retraction database (<http://retractiondatabase.org/>) as well as through Zotero which automatically flags retracted articles. As such, the reference list is unchanged from the original submission. We have included additional references for some of the reviewer 2s comments, and these can be seen in the tracked changes manuscript. Please let us know if there is a specific article in question and we will address this.

**Reviewer #1: Thank you for submitting this good work to Plos one. The paper is well written, and the data analysis is sound and sufficient. I do not have additional comments except that the resolution of Figs in the system was a bit low.**

Thank you so much for your time reviewing this article. We greatly appreciate the time you spent reading the manuscript, and that you found the analysis sound and sufficient. Thank you for your comment on the resolution of the figures being too low, we have resubmitted the figures through PACE and they meet the figure requirements for PLOS ONE.

**Reviewer #2: The manuscript entitled “Different disease inoculations cause common responses of the host immune system and prokaryotic component of the microbiome in Acropora palmata” represents a thorough and needed investigation of host transcriptomic and microbiome responses to distinct disease challenges in a critically important reef-building species. I find the main strength of the paper to be the evidence and discussion of microbiome succession during early stages of coral disease. Most coral disease studies focus entirely on corals that already have advanced signs of disease and have very likely progressed past the microbial determinants and host responses to the initial causative event. My main criticism with the paper involves the heat stress component, which is ill-defined in the context of environmental variation for these corals and the results of which are confusingly reported. Specific recommendations for this limitation, as well as other recommendations to improve clarity and better inform readers of methodologies used, are described below. Lastly, I found the manuscript to be rather overwhelming in length and supplements. I appreciate the thorough analysis and transparent reporting, but I encourage the authors to take advantage of any opportunities to minimize redundancy and be more concise.**

Thank you so much for your in-depth review of the manuscript, we really appreciate the time you have spent giving suggestions and feedback, and we will be addressing all your comments below to improve the manuscript. We also appreciate your comment on the length. Originally, the manuscript body (excluding references) was 90 pages long and was substantially refined to the current length of 40 pages which we believe provides adequate detail and analysis for the large amount of data generated. We will be going back through to try to reduce redundancy and improve conciseness where relevant.

Please find below answers to all your identified comments, with the updated relevant sections from the manuscript also provided and underlined.

**Methods:**

**Comment:** Line 125: Please specify how many ramets of each genet. Alternatively, this information could be included in the tank descriptions.

**Response:** Thank you for identifying that it was unclear the number of ramets in each genet. The field collected fragments of the four genets were larger (11cm by 11cm, roughly 32 per genet) and underwent fragmentation to produce 40 fragments of each genet for the experiment (6cm by 3cm). We have clarified this in the methods and have also added the number of fragments of each genet used in the STHS sections, and the disease inoculations.

**Line 130:** Roughly 32 fragments (11cm by 11cm) of four genets (HS1, CN2, ML2, and CN4) of *A. palmata* were collected in July 2019 from the Coral Restoration Foundation’s (CRF) offshore nursery in Key Largo, Florida, and transported to four raceways in the Experimental Reef Lab (system described in (77)) in Miami, FL.
**Line 140**: Fragmentation yielded 40 fragments of each genet to be used in the experiment, with recovery from fragmentation occurring for three weeks.

**Line 151:** identified for bleaching to occur. Allocation to temperature treatments resulted in 20 fragments of each genet receiving either the ambient, or STHS, temperature treatment.

**Line 207:** (Thermofisher, MA) and stored at -80^o^C. For each temperature treatment (ambient or STHS), this resulted in five fragments of each genet receiving one of the four disease inoculations.

**Comment:** Line 138: How do the 27C control and 30C heat stress compare to minimum-average-maximum temperatures these corals experience in the nursery in these same months? Relative to seasonal variation and SSTs during known bleaching events, is 30C a light, moderate, or severe heat stress?

**Response:** Thank you for your comment about the severity of the heat stress used. Unfortunately, there is no data available for the nursery for us to draw direct conclusions about what the corals experienced during this time. The ambient temperature was set as the temperature the day the corals were collected from the nursery, and thus the same/similar to what these corals are probably experiencing at the time of collection. We have updated the methods stating this.

**Line 137:** each raceway. Tank water temperature was set to 27.5^o^C following temperature readings at depth within the coral nursery, with this also set as the ambient temperature treatment.

For your second point, for reefs in the Florida Keys, bleaching is predicted to occur when daily average temperatures are “≥30.5 °C for 16 days, or 1 day ≥31 °C” (Manzello *et al*, 2007). As the short-term heat stress used is below this temperature and time duration, we are classifying it as a light heat stress for the corals.

**Line 147:** days at 30^º^C, 5-day ramp down to 27.5^º^C (-0.5^º^C d^-1^). The 30 ^o^C was classified as a STHS due to reefs in the Florida Keys being predicted to bleach when average temperatures are >30 ^o^C for 16 days (66), and *A. palmata* showing increased bleaching incidence after 10 days at 31 ^o^C (78). Therefore, the STHS at 30 ^o^C for five days was chosen as it was below the temperature and time thresholds identified for bleaching to occur. Allocation to temperature treatments resulted in 20 fragments of each genet receiving either the ambient, or STHS, temperature treatment.

**Comment:** Line 153: Did the authors prepare WBTi DS twice (once for each inoculation) or did they preserve it between inoculations (or something else)? Please clarify.

**Response:** Thank you for identifying that the WBTi DS preparation was not clear in the manuscript. The WBTi DS was prepared fresh for each of the two inoculations that were administered. We have added an additional sentence at the end of the WBTi DS preparation section clarifying this.

**Line 172**: A total of 20 ml of the diluted slurry was added to each jar, using a 50 ml serological pipette (28). The day-0 and day-3 inoculations were both prepared on their respective day using different fragments of diseased *A. cervicornis* fragments.

**Comment:** Line 165: The same healthy fragments were used to generate HTS: does this mean that some slurry was removed from a ramet one day and then more tissue was removed another day? Or does this mean that slurry was removed from a ramet and then more tissue was removed from another ramet of the same genet for the next inoculation? Or does this mean that tissue was removed once and preserved between inoculation? Or something else? Please clarify.

**Response:** Thank you for again identifying that the HTS preparation was not clear. The HTS used the same donor fragments for the two inoculations (i.e., for the branches of *Acropora cervicornis* used for the HTS, half the tissue from the used fragments was removed for the Day-0 inoculation, and the other half for the Day-3 inoculation). We have updated the manuscript accordingly.

**Line 181**: of *A. cervicornis* were used to generate the Day-0 and Day-3 inoculations, with half of each fragment’s tissue removed on day-0, and the other half removed on day-3.

**Comment:** Line 174: Was this process repeated for the second inoculation? If so, how did the authors ensure consistency in inoculation loads between doses (e.g., plating out/measuring OD/calculating CFU/mL?) Please clarify.

**Response:** Thank you for again identifying that it was unclear whether a new inoculation was prepared, or an old inoculation was used for the day-3 dose of *SM*.

Firstly, we prepared a new *SM* inoculation for the day-3 inoculation, and we have provided this clarification in the text.

Secondly, we did not undertake any of the identified metrics to ensure consistency in inoculation between the two *SM* inoculations, which we acknowledge was an oversight and will be included in any future experiments using *S. marcescens* and this methodology. While we cannot identify the inoculation loads, there was successful amplification of triplicate replicates of the day-0 and day-3 *SM* inoculations showing successful culture and inoculation of *S*. *marcescens*. Additionally, the 16S rRNA analysis showed similar read depths and abundances between the day-0 and day-3 *SM* inoculations. We again acknowledge that this is not a rigorous methodology (as per your suggested metrics), but it indicates that the inoculation loads were most likely not that different.

**Line 191**: administered directly to live *A. palmata* fragments in their jars. Fresh *SM* inoculations were generated for the day-0 and day-3 inoculations.

**Comment:** Line 189: Explain the coral sampling procedure (e.g., approximate size of tissue removed), including how any lesion produced by the sampling may have affected coral health in subsequent exposures.

**Response:** Thank you for identifying these omissions concerning our tissue sampling. We have now added the following text to the methods section clarifying the sample size:

**Line 206**: All tissue samples taken were roughly 0.5cm by 0.5cm, placed in 1.5ml of RNAlater (Thermofisher, MA) in 2ml cryovial tubes, and then stored at -80^º^C.

We have also added the following text to the discussion section addressing the potential for the lesion to influence infection following inoculation.

**Line 927**: of the whole coral holobiont. It is also important to acknowledge that the pre-exposure sampling of the corals could have contributed to increased mortality or disease signs via the resulting wound. We observed, however, little to no disease or mortality in corals that received the healthy inoculations (HTS or *S*P) indicating that disease manifestation was indeed due to the pathogenic inoculations administered (WBTi DS or *SM*). Our results should still be interpreted conservatively in the context of the relative treatment effects and not as a representation of true transmission rates observed in the field. Future work following similar methodologies should be conducted with a subset of control corals receiving no inoculation after sampling, thereby identifying any potential detrimental effects due the sampling itself.

**Comment:** Line 240: Please clarify the basis of enrichment. Only significant genes were used as input, and this was run against the total list of genes? Or was the enrichment based on p-value within the list of significant genes?

**Comment:** Line 251: Similar as above, please clarify the basis of enrichment. For example, within-module genes were input run against all other genes? Or was some continuous value (e.g., kME?) used as the basis of enrichment.

**Response:** Thank you for identifying the confusion with the GO and KEGG enrichment criterion, We have provided the edits to the manuscripts for both of these points below. Inputs for enrichment tests were dependent on analysis methods, so therefore it was either significant genes identified through DeSeq2, or the genes within modules identified from WGCNA. The identified gene sets (be it significant genes from DeSeq2 or all genes in a WGCNA module of interest) were then tested for GO enrichment against the genes in the *A. palmata* genome that were retained for analysis following low count removal, or the KEGG orthology database.

In trying to be more concise, we decided to state, for each analysis, the gene sets which would be used for GO and KEGG enrichment tests, and then provide the specific methods for the enrichment tests at the end of this section. We have provided some additional clarification in text about the inputs for the enrichment tests but have left the overall structure the same for this section. Please let us know if this is still confusing or if we have misunderstood your comment. We have also underlined in the enrichment sections where we state the background set that interesting sets of genes were tested against.

**Line 259**: *Exposure + Genet*) and reduced model (*~ Genet*). All significant genes identified from the LRT analysis (p-adj < 0.001) were used as input for Gene Ontology (GO) and Kyoto Encyclopedia of Genes and Genomes (KEGG) pathway enrichment analysis.

**Line 269**: was done using Complex Heatmap (93). All genes within modules were used as input for subsequent KEGG pathway and GO enrichment analyses. Significant GO and KEGG pathways were used to infer each module’s putative function.

**Line 272**: GO enrichment analysis was run using Cytoscape v3.8.2 (94) and BiNGO v3.0.3 (95) using the GO to gene identifiers available in the *A. palmata* genome annotation file (85,86). The hypergeometric test was utilized, and *p*-values were corrected with a Benjamini & Hochberg false discovery rate (FDR) with alpha set at 0.01. The genes remaining in the *A. palmata* genome after low count filtering were used as the background set for enrichment testing. Visualization of GO enrichment was undertaken in Cytoscape to identify relationships between significantly enriched child and parent terms. Genes present in significantly enriched terms were then visualized using the VST counts and Complex Heatmap (93).

**Line 280**: KEGG enrichment was run using the gene to KEGG ID’s available in the *A. palmata* genome annotation file (85,86) and the R package clusterprofiler v3.18.1 (96). Enrichment was tested against the KEGG Orthology database (organism = ko) with alpha set at 0.01. Visualization of enriched pathways was undertaken in clusterprofiler (96), enrichPlot 1.10.2 (97), and GGPlot2 (98).

**Comment:** Line 310: The module-on-module correlation described here is complicated and could use another sentence or two for clarification. If I’m understanding correctly, each sample has a continuous eigengene value for each module, and those values are correlated? I think recall per-sample MEs but the generic WGCNA description of ME as the first PC of the module lends the impression of a single ME per module, which wouldn’t facilitate correlation analysis.

**Response:** Thank you for identifying that the module-on-module correlation is confusing how it was originally stated. You are correct that each sample has a continuous eigengene for each module, and that these values, for the modules of interest, were correlated together. You are also correct that the first module eigengene represents the first principal component, but it is as a eigen vector. As such the module eigengene contains a single value for each sample within the data set, and as such is a multi-value vector that allows correlation among one another (as well as against metadata as is done in the normal WGCNA analyses). We have provided this clarification in the manuscript.

**Line 328**: were run using the module eigengenes from each analysis. The first module eigengene represents the first principal component of a module as an eigen vector, which therefore results in a value for each sample used in analysis. Thus, these multi value vectors can be correlated against one another allowing correlative analysis between the different omics WGCNA analyses.

**Comment:** Line 361: I’m not sure how to interpret this sentence. “…when splitting these six modules by the four disease inoculations” – does this mean when looking at the correlations of each module with each of the four disease inoculations? When I do that, I see that the directionality of correlation is the same across disease inoculations for these six modules (where shown). But then “all correlations for visually healthy and diseased coral samples showed the same correlation directionality” isn’t true for these six modules. Blue, Black, and Dark Green have opposite signs between VH and D. Please clarify.

**Response:** Thank you for identifying the confusion with the wording of this sentence. We have updated the manuscript text to reflect the intended point that the correlation direction is the same when split by the disease inoculations within the visually healthy corals, and the same when split by the disease inoculations for diseased corals.

**Line 408**: visually healthy, and diseased coral samples (Fig 2C). There was no difference in module correlation directionality when splitting the visually healthy corals by their respective disease inoculation (WBTi DS, HTS, *SM*, *S*P) compared to all visually healthy samples grouped together (Fig 2C). Similarly, diseased corals split by disease inoculation (WBTi DS & *SM*) also showed the same module correlation directionality when compared to all diseased samples grouped together (Fig 2C).

**Results:**

**Comment:** **Line 322:** The methods only describe alignment to the genome. Why is the alignment rate to the transcriptome mentioned here? Are the alignments to the transcriptome used in the analysis?

**Response:** Thank you for catching this inconsistency in the text of what was aligned. For the pre-processing, we aligned to the genome and the transcriptome. All downstream analysis was run with the files that were aligned to the transcriptome. We have clarified this in the text and provided this updated section below.

**Line 249**: and transcriptome (85,86). Downstream analysis utilized the reads aligned to the reference transcriptome.

**Line 341**: to the *A. palmata* transcriptome, and 66% alignment to the *A. palmata* genome (85,86). For downstream analysis, the samples aligned to the transcriptome were used.

**Comment:** Line 356: I think “2A” and “2B” are switched in this sentence. Figure 2A shows groupings by inoculation and Figure 2B shows groupings by visual status. Also, both appear to show substantial separation by inoculation or visual status. Please provide statistics to support the statement that analysis of variance did not find significant differences between disease inoculations. It would be helpful if those stats could be provided on the figures directly as well.

**Response:** Thank you for catching this mistake. The intext reference and figure caption are correct, and we have fixed Fig 2 which had A and B switched.

For the second request, please find PERMANOVAs run between the visual health status (pre-exposure, visually healthy, and diseased) as well split by disease inoculations within the visually healthy samples (WBTi DS, HTS, *SM*, and *S*P) and diseased samples (WBTi DS and *SM*). We have also updated the methods section. We have chosen not to include the stats on the figures as it made the plots extremely busy and confusing. We hope having the stats included in text is an acceptable compromise.

**Line 254**: Permutational multivariate analysis of variance (PERMANOVA) were run using vegan (89) on the PCA and metadata variables of interest using the VST, and batch removed VSTs as input. For multilevel factors, the initial PERMANOVA was run with a subsequent pairwise comparison (90).

**Line 400**: were used (Fig 2B). PERMANOVA analysis corroborated this result with significant differences between pre-exposure and visually healthy (p-adj = 0.03, R^2^ = 0.07), pre-exposure and diseased (p-adj = 0.03, R^2^ = 0.11), and visually healthy and diseased (p-adj = 0.03, R^2^ = 0.17) coral samples, but no significant differences between the disease inoculations within the visually healthy and diseased corals.

**Comment:** Line 429: Was any manual characterization of this predicted/uncharacterized gene attempted? E.g., manual BLAST to check for updated annotations or protein domain prediction.

**Response:** For this analysis, there was no manual BLAST run for any of the hypothetical/uncharacterized genes. The annotation file used for the analysis was recently updated by the authors who generated the *Acropora palmata* genome, and as such we did not provide any additional characterization/annotation. For future work with this genome, this is 100% something that we will be incorporating so to make sure the most up to date annotations and protein domain predictions are present.

**Comment:** Line 449: “regardless of which disease inoculations was administered (Fig 3A)” but Fig 3A shows some separation grouping by health status. Please provide stats.

**Comment:** Line 450: “with no unique profiles identified due to the four disease inoculations (Fig 3B)”, but again, Fig 3B does seem to show some clustering. Please provide ANOVA or similar stats for these analyses.

**Response:** Thank you for these comments in regards to the principal component analysis of the 16S rRNA data and the lack of statistical analysis. We have updated this section to include PERMANOVA analysis which provides the statistical backing of these statements.

**Line 494**: PCA analysis identified coral samples grouping by VHS (Fig 3A) with no unique profiles identified within the visually healthy or diseased corals due disease inoculation (Fig 3B). PERMANOVA analysis corroborated this result with their being significant differences between pre-exposure and visually healthy (p-adj = 0.03, R^2^ = 0.104), pre-exposure and diseased (p-adj = 0.03, R^2^ = 0.111) and visually healthy and diseased corals (p-adj = 0.03, R^2^ = 0.079). There were, however, no significant differences within the visually healthy or diseased corals due to the different disease inoculations administered.

**Comment:** Line 458: The Shannon value stated in the text (2.25) doesn’t match the value shown in 3C (looks very similar to the VH value). The 2.25 value would be significantly less than the 4.52 value, but the values shown on the figure look similar. Please double-check stats here and make sure you’re reporting consistent values.

**Response:** Thank you very much for catching this error in text. We have rerun the analysis to double check, and the values plotted in the figure are correct. We have now corrected the in-text error, as well as adding in the Shannon values for the pre-exposure and visually healthy corals.

**Line 508**: corals were significantly more diverse (Shannon-Weiner = 4.24) compared to pre-exposure corals (Shannon-Weiner = 3.78**,** *p* < 0.01), but less diverse than visually healthy corals (Shannon-Weiner = 4.53**,** p < 0.01; Fig 3C).

**Comment:** Line 572: The description of this section only references transcriptomic and 16S results, but it also includes all the heat stress results, disease outcomes, buoyant weight, and PAM data. I recommend restructuring for clarity.

**Response:** Thank you very much for catching this error. The sub results heading was deleted during the final editing before submission. We have rectified this so that the results sections now have their respective headers.

## Line 469: The short-term heat stress did not affect physiological metrices and elicited a weak response from the coral host and prokaryiome.

**Comment:** Line 605: I think the heat stress results could be moved sooner. I’m guessing the lack of temperature effect is why the earlier sections of the results didn’t include any analysis of temperature. It’s unclear why these results are included in the current section, and up until this point, I had been wondering what happened to heat treatment and what the differences in disease outcome were between groups.

**Response:** Thank you very much for your comment on this. We elected to put the temperature results later on in the manuscript as there was no strong effect of the treatment. To address your concern, we have reordered the results section, putting the temperature results after the 16S rRNA analysis of the disease inoculations.

**Line 372: The short-term heat stress did not affect physiological metrices and elicited a weak response from the coral host and prokaryiome.**

There were no significant differences (alpha<0.05) in I-PAM (S6 Fig B) or buoyant weight (S6 Fig C) measurements between ambient or STHS treatments. There were also no significant increases in risk of developing disease signs due to temperature treatment and disease inoculations within each genet (S7 Fig A) or between the genets (S7 Fig C). When evaluating disease inoculations independent of temperature, there was a significant difference in risk of disease signs developing between the *Serratia* RR and WBTi RR for only genet ML2 (S7 Fig B).

For the coral hosts transcriptomic response, PCA did not identify any clear separation in the 95% confidence intervals for all (S8 Fig A), pre-exposure (S8 Fig B) or disease inoculated (S8 Fig C) coral samples. Differential expression analysis identified 28 significantly differentially expressed genes (DEG) for the pre-exposure coral samples (S9 File) with hierarchical clustering of these genes causing separation between ambient and STHS coral samples (S8 Fig D). For disease inoculated samples, there were 21 significant DEG which all showed positive log 2-fold changes (L2FC) (S9 File) but no clear clustering between ambient and the STHS (S8 Fig E).

PCA of the prokaryiome did not identify any clear separation of 95% confidence intervals for all (S9 Fig A), pre-exposure (S9 Fig B), or disease inoculated (S9 Fig C) corals. PERMANOVA analysis did however identify a significant difference between the compositions of ambient and STHS (*p* < 0.001) corals. Differential abundance analysis identified eight ASVs with significantly increased abundance, and nine ASVs with significantly decreased abundance in the pre-exposure STHS coral samples (S10 File). Alpha-diversity analysis of the pre-exposure coral samples identified significant differences in the Shannon-Wiener (p < 0.01) estimates with STHS coral samples showing a more diverse prokaryiome than ambient corals (S9 Fig E).

**Figures:**

**Comment:** Figure 2: A and B are switched.

**Response:** Thank you for catching this mistake, this has been rectified. Please see below for the updated Figure 2 with A and B in the correct order which now matches the in text references and figure caption.

**Comment:** Figure 5: The simplified layout of the main correlative heatmap didn’t add anything for me. I spent a few moments trying to figure out why those boxes were empty before I realized what was happening. I think the layout of the main heatmap is fine, given that it has the separation between the left and right parts with descriptive labels above them. One could simply add the words “modules” to the top labels and a “Transcriptomic modules” (maybe “Coral transcriptomic modules” to be extra clear) label along the y-axis to completely recapitulate the “simplified” diagram.

**Response:** Thank you very much for this suggestion. Please see below the updated Figure 5 in which we have removed the simplified layout, and incorporated the labeling schema that was suggested.

**Comment:** S6: This figure could be a good place to include some sort of seasonal data trends layered on top of tank measurements. E.g., average min/max values for July/August over the last few years as additional lines on S6A.

**Response:** Thank you for your comment about inclusion of additional data to figure S6. We agree that field data linking these experiments to the nurseries where the corals were originally grown would be helpful. Unfortunately there is no in-situ water temperature data available for this coral nursery. To address this point we have stated in the methods that the ambient temperature was set to 27.5C as this was the temperature recorded in the nursery when fragments were collected for the experiment.

**Line: 13**: each raceway. Tank water temperature was set to 27.5^o^C following temperature readings at depth within the coral nursery, with this also set as the ambient temperature treatment. Following

**Discussion:**

**Comment:** Line 824: This caveat is important and would be a good place to reference normal seasonal values for this species in situ. Has paling or any other signs of stress been observed for this species at 30C for ~15 days? How normal is it for them to experience this level of temperature stress?

**Response:** Thank you for identifying the lack of information on normal seasonal values for *A. palmata* in situ. We have added in additional information in the discussion addressing this point.

**Line 844**: enough, to elicit the hypothesized increase in disease susceptibility. In-situ, *A. palmata* has been shown to show increased bleaching incidence with cumulative heat stress at 31 ^o^C for greater than 10-days (78). This indicates that the strength and duration of the STHS used here was not enough to cause the early transcriptomic changes associated with the bleaching response, and thus cause no changes in disease susceptibility. Additionally, previous work in *A. palmata* identified clear transcriptomic profiles at a two day STHS of 32 ^o^C (151). While the STHS used in this study was longer than the previous work in *A. palmata* (151), 30^o^C may have been too low to initiate and cause the previously identified transcriptomic profiles.

**Comment:** Line 826: What was the duration and magnitude of short-term heat stress in this cited study that did find clear transcriptomic responses to STHS? How does that compare to the parameters in this study?

**Response:** Thank you for identifying that the comparison to the prior short term heat stress study was not evident in the discussion body. We have updated this section with the relevant information.

**Line 848**: susceptibility. Additionally, previous work in *A. palmata* identified clear transcriptomic profiles at a two-day STHS of 32 ^o^C (151). While the STHS used in this study was longer than the previous work in *A. palmata* (151), 30^o^C may have been too low to initiate and cause the previously identified transcriptomic profiles.

**Comment:** Line 835: One can theoretically agree with the premise of this section (that differences in baseline immune expression drive differences in susceptibility), but this study doesn’t provide any supporting evidence. You observed the differences in immune gene expression, but not the differences in susceptibility (S7C). The last sentence (Line 847–848) is not supported. Please rephrase or clarify the connection between this hypothesis and the observations of this study. E.g., why didn’t you see that genets with higher immune expression having lower rates of disease?

**Response:** Thank you for your comment and suggestions on this point. We do agree with you that our study does not provide clear evidence for this point, and have rectified the wording of this section as an interesting observation that should be explored and further researched using a larger number of genets of *A. palmata* in a disease exposure experiment.

**Line 871**: pathogen-associated molecular patterns (PAMPs) (155,156) indicating that higher baseline expression of these pathways could confer increased disease resistance due to a primed and active immune system. It is important to note that while we did observe differences in baseline immune expression between the four genets, there were no significant differences in disease susceptibility identified. We therefore recommended that this observation be built upon utilizing a larger set of genets of *A. palmata* in a disease exposure study so to identify if 1) differences of baseline immune expression hold true in a larger sample size, and 2) if the differences of baseline immune expression do correlate and explain differences in disease susceptibility and resistance.

**Comment:** Line 889: I don’t understand this line: “inclusion of experimental corals not exhibiting any inoculations.” I think this is a suggestion to include control (unchallenged) corals in experiments, but I’m not sure given the “exhibiting inoculations” language. Please clarify.

**Response:** Thank you for identifying the confusing terminology here. You are correct in your comment on what was intended to be conveyed, and we have improved the phrasing to make this more concise.

**Line 1099**: specialized responses to different types of pathogenic organisms. Inclusion of control corals without any homogenate inoculation would also be an important consideration for future work, as this would allow identification of shifts in the coral hosts transcriptomic response, and prokaryiomes response, caused by control inoculations (HTS and *S*P). Additionally,

**Comment:** Line 901: By including observational temperature data, one could make a more informed recommendation here than simply “wider range of temperatures.” For example, the recommendation could be to design experiments around observed and predicted temperature models for these species/populations.

**Response:** Thank you for your comment about including observational temperature data. Unfortunately there is no in situ nursery temperature data available, and as such it was not possible to include this. We have, however, rephrased this section to make the recommendation of using observed and modeled temperature scenarios to design future experiments like this for *A. palamta*, as well as other coral species.

**Line 941**: are reached. Specifically, tailoring heat stress intensity and duration based on historical data for the location of collection of the corals, as well as relevant thresholds that are present in the scientific literature. This will allow identification of potential tipping points before the bleaching threshold is reached, or whether active bleaching signs are this tipping point, and required for a loss in disease resistance. Future work should also look to test predicted and modeled future temperature scenarios which will allow identification of the impacts of disease exposure on the performance of coral species under different climate change scenarios. Incorporation of a wider range of genets would also be insightful to catch heat tolerance variability that is within the wider *A. palmata* population.

**Comment:** Line 915: Do the authors expect to find a similar lack of a “strong signal of genet identity” in prokaryiome communities in wild A. palmata? I could imagine an expectation of more similar microbiota among members of a restoration population than among “rare outcrops” unless the authors have additional citations to support the core prokaryiome across this species’ range. If the authors agree, maybe add a clarifying statement here or in earlier discussions of the “core prokaryiome” to address the possibility that this finding is specific to nursery corals.

**Response:** Thank you for your comment about the core microbiome of *A. palmata* between restoration and rare wild outcrops. We have updated our conclusions with a clarifying statement to include your mentioned point.

**Line 961**: An important caveat to note here however is that the majority of 16S rRNA work for *A. palmata* has been carried out on nursery-based corals, and thus the core microbiome observed may only be present in nursery reared corals. Additional research to identify whether the core microbiome identified in nursery reared *A. palmata* is similar/different to fully wild *A. palmata* colonies should be undertaken, as this would allow identification of whether the prokaryiome does hold true over space and time. Identifying similarities in the prokaryiome between fully wild and nursery corals would also provide management benefits, as probiotic treatments developed from nursery corals could be used in wild outcrops. Finally, our results provide evidence that focusing

**Minor comments:**

**No action needed: I’m just curious if the authors are inventing the term “prokaryiome” here. I’ve never heard it and a quick google search yielded nothing. I personally would accept an original description of the “bacterial and archaeal microbiome” and then subsequent use of simply “microbiome.” I know a small but vocal contingent of microbiologists who are trying to retire the word “prokaryote” in favor of “bacteria” or “archaea” where appropriate, and who will probably make fussy noises at this new term. But I also appreciate that this study describes a situation where archaea and bacteria are described together with no implication of their evolutionary relationship, and therefore this use seems appropriate for “prokaryote” to distinguish the two groups as “non-eukaryotic.”.**

Thank you for your comments on the “prokaryiome” term, and yes we did invent it in the manuscript. We would first like to say that being specific shying away from the term prokaryote whenever possible is more stringent, especially when looking at evolutionary perspectives. The reason for including “prokaryiome” here is that we have noticed many publications (including coral and other organisms) that use 16S rRNA sequencing and then say they characterized “the microbiome”. The microbiome consists of all microscopic organisms but the 16S rRNA only characterizes bacteria and archaea. As such, we wanted to be clearer throughout the manuscript in what we had explicitly characterized. In earlier drafts we did have, as you mentioned, that we characterized the “bacterial and archaeal microbiome” with it then subsequently referred to as “microbiome”. We changed this to prokaryiome as it avoided re classifying the definition of the microbiome for the entire manuscript, and instead allowed the use of microbiome in its classical definition, with subsequent inclusion of the prokaryiome in discussion points. We hope this provides more context for the use of this term throughout the manuscript.

**Very minor grammatical comment that I only mention because it considerably impedes understanding in some cases: The authors frequently use “this” without a clear antecedent. For example, what does “this” reference in Line 46? Prokaryotic and eukaryotic life or marine environments or infectious diseases? None of the available antecedents from the prior line seem to fit the description of something that “includes healthy ecosystems” in the way the next line describes. Lines 58 (this prevalence of disease?), 60 (this/these criteria?), 63 (this failure to confidently describe etiology?), 67 (this complexity?) and others are less ambiguous, but I’m still not certain I’m guessing the correct “this” in every case.**

Thank you for this comment on the use of “this”. We have addressed the specific instances mentioned above in the manuscript and copied the corrections below. We have also gone through the manuscript and corrected any additional instances of “this” which are not clear.

- **Line 50**: eukaryotic life (1). In healthy ecosystems, low levels of disease can help structure biological communities and be considered part of a balanced ecosystem by influencing food webs and maintaining stable populations (2–4).
- **Line 62**: Despite research efforts over the last 40 years, identification of the causative pathogen(s) of coral diseases has been challenging (15,16).
- **Line 67:** not been possible for many coral diseases due to many marine prokaryotes being unknown and unculturable (18,19), causative agents being consortiums of microbes (20), or diseases being linked to abiotic factors (21–24).
- **Line 71:** archaea, viruses, fungi, and microeukaryotes (25). Due to the complex assemblage of microbiome members in the coral holobiont, it has been difficult to discern between beneficial and detrimental organisms, with putative pathogens also commonly found in healthy coral microbiomes (13,16,26,27).

**Comment:** Line 311: “run-on” can be “run on”

**Response:** Thank you for catching this, this has been corrected in the manuscript text.

**Line 332**: analysis of module eigengenes was only run on samples that had complimentary transcriptomic and 16S rRNA data available.

**Comment:** Line 800: “omics” was previously stylized as “’ ’omics”

**Response**: Thank you for catching this error. We have made sure all instances of omics are as “omics” and not “’omics” in the manuscript.
